# Probing mutation-induced conformational transformation of the GTP/M-RAS complex through Gaussian accelerated molecular dynamics simulations

**DOI:** 10.1080/14756366.2023.2195995

**Published:** 2023-04-14

**Authors:** Huayin Bao, Wei Wang, Haibo Sun, Jianzhong Chen

**Affiliations:** a School of Pharmacy, Shandong University of Traditional Chinese Medicine, Jinan, China; b School of Science, Shandong Jiaotong University, Jinan, China

**Keywords:** M-RAS GTP Gaussian accelerated molecular dynamics free energy landscapes principal component analysis

## Abstract

Mutations highly affect the structural flexibility of two switch domains in M-RAS considered an important target of anticancer drug design. Gaussian accelerated molecular dynamics (GaMD) simulations were applied to probe the effect of mutations P40D, D41E, and P40D/D41E/L51R on the conformational transition of the switch domains from the GTP-bound M-RAS. The analyses of free energy landscapes (FELs) not only reveal that three mutations induce less energetic states than the wild-type (WT) M-RAS but also verify that the switch domains are extremely disordered. Principal component analysis (PCA) and dynamics analysis suggest that three mutations greatly affect collective motions and structural flexibility of the switch domains that mostly overlap with binding regions of M-RAS to its effectors, which in turn disturbs the activity of M-RAS. The analyses of the interaction network between GTP and M-RAS show that the high instability in hydrogen bonding interactions (HBIs) of GTP with residue 41 and Y42 in the switch domain I drives the disordered states of the switch domains. This work is expected to provide a molecular mechanism for deeply understanding the function of M-RAS and future drug design towards the treatment of cancers.

## Graphical Abstract

**Figure:**
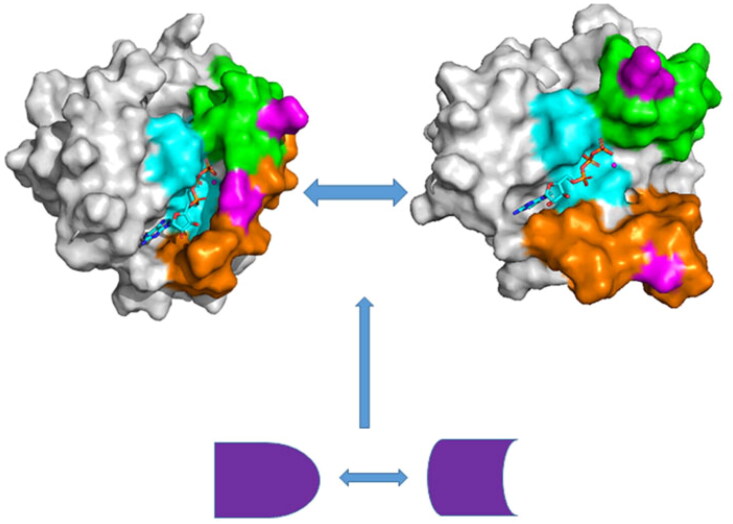

## Introduction

RAS family of small GTPases, mainly including H-, K-, and N-RAS, is the product of the RAS proto-oncogenes. In addition, RAS family of small GTPases is also involved in a number of its relatives, such as Rap1, Rap2, R-RAS, M-RAS, Ral, Rin, Rit, Rheb, etc. These RAS proteins function as a molecular switch by cycling between an active state bound by guanosine triphosphate (GTP) and an inactive state bound by guanosine diphosphate (GDP) in various intracellular signalling pathways regulating cell growth, differentiation, and apoptosis^1–7^. The hydrolysis reaction of GTP into GDP is accelerated by the GTPase activating proteins (GAPs), which yields an inactive form of the GDP-bound RAS proteins^8^. The transformation of GDP into GTP is catalysed by guanosine nucleotide exchange factors (GNEFs), resulting in an active state of the GTP-bound RAS proteins^7–9^. Recently, increasing attentions are paid to these RAS proteins due to their close relations with a variety of human cancers^10–13^. More previous works suggested that mutation-induced activity of RAS proteins has been frequently detected in cancer patients and some small molecule inhibitors have been designed towards treatments of human cancers^14–18^. Therefore further probing possible factors of RAS proteins involved in human cancers are of high significance for drug design towards RAS proteins.

**Figure 1.**
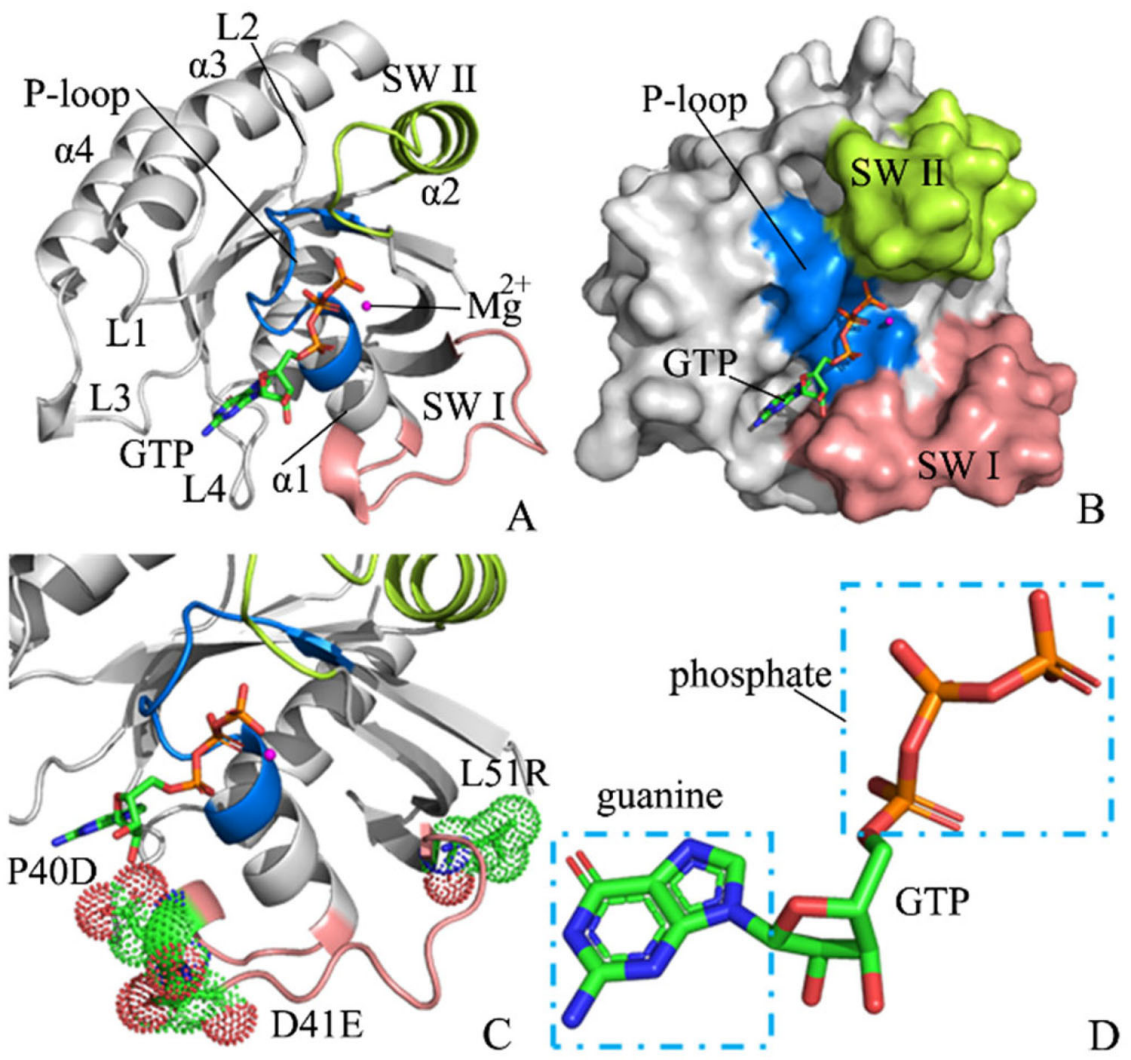
Molecular structures: (A) the GTP/WT M-RAS complex, in which M-RAS, GTP and magnesium ion were depicted in cartoon, stick and ball modes, respectively, (B) binding pocket of GTP to M-RAS, in which binding pocket was shown in surface modes, (C) mutated sites of M-RAS displayed in dot modes (D) the structures of GTP shown in stick styles.

Although H-RAS, K-RAS, N-RAS, and the other relatives of RAS proteins have difference in residue sequence, they share highly similar structural topologies^19–22^ (Figure 1(A)). X-ray crystallographic and NMR analyses of two RAS proteins H-RAS and Rap1A, free or complexed with their effectors, indicated that the exchange of GTP for GDP leads to obvious allosteric conformational alterations in the switch domains, namely SW I (residues 27–40) and SW II (residues 59–75), and makes RAS perform downstream signalling by means of direct interaction with its effectors, such as Raf kinases and phosphoinositide 3-kinase^23^^,^^24^. More importantly, the SW I mostly overlaps with the effector region and produces a main interface of effector identification^25^^,^^26^. On the other hand, although the SW II takes part in a limited role in the effector identification, it is responsible for main interactions with GNEFs and GAPs^24^^,^^27^. Structurally, two switch domains SW I and SW II are involved in the binding regions of GNEFs and effectors (Figure 1(B)), hence the transformation of the states in the switch domains affects the activity of RAS proteins^28–32^. Based on this fact, it is importance to explore molecular mechanism with regard to the conformational transformation of the switch domains for deeply understanding the roles of RAS proteins in cancer treatment.

M-RAS, also termed R-RAS3, shares 60–75% sequence identity of its N-terminal 110 residues and the identical effector region with RAS proteins^27^. The switch domain SW I of M-RAS includes the residues 37–50, while the switch domain SW II is composed of the residues 69–85. Overexpression of the Q71L mutated M-RAS results in cellular transformation or inhibition of differentiation^33^. Despite its weak interactions with some of the RAS effectors, M-RAS leads to weak activation of the downstream cascades^34^^,^^35^. The first tertiary structure of state 1 for the GppNHp/M-RAS complex crystalised by Ye et al. revealed that the loss of the direct and Mg^2+^-coordinated indirect interactions with T45 from M-RAS and the γ-phosphate of GppNHp in the state 2 leads to obvious deviation of the loop in the SW I away from the guanine nucleotide and conformational instability of the loop from the SW I^27^. Moreover, the conformational transformation of the switch domains in M-RAS caused by mutations or environment changes has been involved in human oncogene^17^^,^^36–39^. Little is known about the molecular mechanism underlying conformational changes of the switch domains in M-RAS. Thus it is highly requisite to clarify conformational changes of M-RAS caused by mutations for further insights into the function of M-RAS and drug design targeting cancer treatment.

Conventional all-atom molecular dynamics (cAAMD) simulations show great potential in probing conformational alterations of targets due to inhibitor binding and residue mutations^40–47^. Meanwhile, post-processing analyses based on trajectories of cAAMD simulations also contribute useful theoretical aids to insights into inhibitor-target recognition mechanism^43^^,^^48–54^. Despite the great successes of cAAMD simulations in probing conformational alterations of targets, the conformations sampled by cAAMD simulations are possibly trapped at a locally minimal space, which brings the possibility of insufficient conformational sampling on targets. To avoid this issue, accelerated molecular dynamics (aMD)^55^ and GaMD simulations^56–58^ were proposed to enhance the sampling efficiency of targets. It is worth noting that these two simulation technologies have been widely used to decipher inhibitor-target binding modes and mutation-induced conformational alterations of targets^41^^,^^59–62^, including work on insights into mutation-mediated influences on the GTP- and GDP-bound K-RAS performed by Chen et al.^9^. Compared to Chen’s work, this study mainly focuses on exploration on effect of mutations occurring at the switch domain SW I on the conformation alterations of M-RAS, a RAS protein that is not widely studied, which can provide useful supplements for deeply understanding the function of RAS proteins. Thus, GaMD simulations provide a reliable approach for further detecting conformational alterations of M-RAS induced by mutations.

In order to achieve our expectation to probe mutation-mediated impacts on conformations of M-RAS, the wild-type (WT), P40D, D41E, and P40D/D41E/L51R M-RAS bound by GTP were chosen to perform our current study. The sites involved in three mutations were depicted in Figure 1(C) and the structure of GTP was shown in Figure 1(D). As shown in Figure 1(C), mutations P40D, D41E, and P40D/D41E/L51R occur at the SW I and they produce significant influences on conformational states of the switch domains, which in turn affects the binding of M-RAS to its effectors^19^^,^^27^. Thus, clarifying the molecular mechanism of mutation-mediated impacts on conformational states of the switch domains of M-RAS is essential. For this current study, GaMD simulations, principal component analysis (PCA)^63–67^, analyses of interaction networks, and construction of free energy landscapes (FELs) were integrated to explore effect of three mutations P40D, D41E and P40D/D41E/L51R on conformational changes of M-RAS. This work is expected to provide a molecular mechanisms and an energetic basis for further understanding the function of M-RAS and its roles in future anti-cancer drug design towards RAS proteins.

## Theory and methods

### Initialisation of systems

The structure of the GTP/M-RAS complex is unavailable in the protein data bank (PDB). The crystal structure of the GppNHp/P40D M-RAS complex (PDB entry: 3KKP)^19^ was superimposed with that of the GTP/K-RAS complex (PDB entry: 5VQ2)^68^, and then GppNHp and K-RAS were removed from this superimposed structure to produce the structure of the GTP/P40D M-RAS complex. To keep the consistence of atomic coordinates, the residue D40 in the GTP/P40D M-RAS complex was changed into P40 to obtain the structure of the GTP/WT M-RAS complex. The similar treatments were carried out to yield the structures of the GTP-bound D41E and GTP-bond P40D/D41E/L51R M-RAS. The protonated states of residues from M-RAS were checked using the web server of the program H++ 3.0 and rational protonation states were assigned to each residue of M-RAS^69^. A magnesium ion (Mg^2+^) was kept at the starting systems. The force field parameters involved in this study were produced with the Leap module in Amber 20^70^^,^^71^ by following the scheme as below: (1) the missing hydrogen atoms in the crystal structure were connected to their heavy atoms, (2) the force field parameters of the WT and mutated M-RAS were obtained by using the *ff*19SB force field^72^, (3) the force field parameters of GTP were taken from the work of Meagher et al.^73^, (4) an octahedral periodic box of water with a buffer of 12.0 Å, consisting of ∼5600 water molecules, was adopted to solve each M-RAS-related complex and the force field parameters of water molecules were assigned by utilising the TIP3P model^74^ and (5) the appropriate number of sodium ions (Na^+^) and chlorine ion (Cl^-^) were placed around each M-RAS-related complex to construct a neutral system in the 0.15 M NaCl salt environment, in which the parameters of Na^+^, Cl^-^, and Mg^2+^ were extracted from the work of Joung and Cheatham^75^^,^^76^.

### GaMD simulations

Initialisation of four M-RAS-related systems possibly results in high energy contacts and orientations between atoms, which causes instability of systems during simulations. To address this unfavourable factor, the GTP-bound WT and mutated M-RAS systems all were subjected to two step minimisation, consisting of a 6000-cycle steepest descent minimisation and a 10000-step conjugate gradient minimisation. The temperature of these four systems was enhanced from 0 to 300 K within 2 ns in the canonical ensemble (NVT), from which all non-hydrogen atoms of the solutes were constrained through a weak harmonic restriction of 2 kcalmol^−1^ Å^2^. After that, a 4-ns equilibrium was performed at 300 K under the isothermal − isobaric ensemble (NPT). Then, a 20-ns NPT simulation was conducted to maintain the density of each system at 1.01 g/cm3. Subsequently, four independent 100-ns cAAMD simulations were implemented at the NVT, in which periodic boundary conditions (PBCs) and the particle mesh Ewald (PME) method were adopted^77^. Finally, four ending structures arising from the above cAAMD simulations were used to conduct four independent GaMD simulations of 1.2 μs with randomised initial atomic velocities.

In our GaMD simulations, a harmonic boost potential was utilised to reduce free energy barriers of four systems and obtain full samplings of conformations. If the potential energy $V(\vec{r})$ of the system is lower than a threshold energy E, $V(\vec{r})$ will be changed into $V^{*}(\vec{r})$ through Equations (1) and (2) as below

(1) $$V^{*}(\vec{r})=V(\vec{r})+\Delta V(\vec{r})$$

(2) ΔV(r⇀)={0, V(r⇀)≥E12k(E−V(r⇀))2,  V(r⇀)<E

through the above equations, the parameter k signifies the harmonic force constant, furthermore, two parameters E and k can be regulated through two following criterions

(3) $$V_{\max}\leq E\leq V_{\min}+\frac{1}{k}$$

(4) $$k=k_{0}\frac{1}{V_{\max-}V_{\min}}$$

where if E is set as the lower bound $E=V_{\max},$ then $k_{0}$ is obtained from Equation (5)

(5) $$k_{0}=\min(1.0,\;\frac{\sigma_{0}}{\sigma_{V}}\cdot \frac{V_{\max}-V_{\min}}{V_{\max}-V_{\mathrm{avg}}})$$

on the contrary, if E is set as the upper bound $E=V_{\min}+\frac{1}{k},$ then $k_{0}$ is derived from Equation (6)

(6) $$k_{0}=(1.0-\frac{\sigma_{0}}{\sigma_{V}})\cdot (\frac{V_{\max}-V_{\min}}{V_{\mathrm{avg}}-V_{\min}})$$

in the above equations, three energies $V_{\max},$ $V_{\min}$ and $V_{\mathrm{avg}}$ represent the maximum, minimum and averaged potential energies of four current systems, individually. The parameters $\sigma_{V}$ and $\sigma_{0}$ are respectively described as a standard deviation of the system potential energy and a user-determined upper limit for accurately reweighting. For our current work, four independent GaMD simulations, each for running 1.2 μs, were implemented on the GTP-bound WT, P40D, D41E, and P40D/D41E/L51R M-RAS, hence the total simulation time of each system is 4.8 μs. Four independent GaMD trajectories were combined into a single GaMD trajectory (SGT) to execute the post-processing analyses. The program PyReweighting stemming from the work of Miao et al.^78^ was wielded to accurately reweight GaMD simulations and detect the original free energy of the WT and mutated M-RAS systems. For all simulations performed in this study, the chemical bonds connecting hydrogen atoms to non-hydrogen atoms were constrained by means of the SHAKE algorithm^79^. The temperatures of the WT and mutated M-RAS systems were tuned with the Langevin thermostat^80^, in which a collision frequency of 2.0 ps^−1^ was used. The PME method with an appropriate cut-off value of 12 Å was utilised to compute electrostatic interactions, and this cut-off was also adopted to estimate the van der Waals interactions. All simulations involved in this study were executed by employing the program pmemd.cuda inlayed in Amber 20^81^^,^^82^.

### Post-processing analyses of GaMD simulations

To check the impacts of three different mutations on structural fluctuations of M-RAS, root-mean-square fluctuations (RMSFs) of the C_α_ atoms from M-RAS were estimated with the SGT. Radius of gyrations (Rgs) and molecular surface areas (MSAs) of M-RAS were also calculated to examine the effect of mutations on the structural compactness of M-RAS. PCA was performed by diagonalising on the covariance matrix constructed with the coordinates of the atoms C_α_ saved in the SGT based on the following equation

(7) $$C=<(q_{i}-<q_{i}>){\left(q_{j}-<q_{j}>\right)}^{T}>$$

where $q_{i}$ and $q_{j}$ respectively represent the Cartesian coordinates of the *i*th and *j*th C_α_ atoms in M-RAS, while $<q_{i}>$ and $<q_{j}>$ denote their averaged positions over conformational ensembles arising from GaMD simulations. The eigenvector and the eigenvalue stemming from the PCA respectively describe collective motions of the structural domains and the fluctuation amplitude of the structural domains along an eigenvector. The details concerning the PCA have been clarified in our previous works^83^.

Meanwhile, dynamics cross-correlation maps (DCCM)^84–86^ were also computed with the coordinates of the C_α_ atoms in M-RAS through Equation (8) to access the influences of three mutations on moving modes of M-RAS.

(8) $$C_{ij}=\frac{<{\Delta r}_{i}\cdot {\Delta r}_{j}>}{{\left(<{\Delta r}_{i}^{2}><{\Delta r}_{j}^{2}>\right)}^{1/2}}$$

in which where ${\Delta r}_{i}$ represents the displacement of the *i*th C_α_ atom away from its averaged position and the angle bracket indicates an ensemble average on the snapshots kept in the SGT. The values of $C_{ij}$ are located at a range from −1 to +1, from which the positive and negative $C_{ij}$ values separately signify the positively correlated motions (PCMs) and anticorrelated movements (ACMs) between residues. Impacts of mutation on moving modes of M-RAS can be efficiently characterised through the changes of $C_{ij}.$ In this work, PCA and calculations of DCCMs were carried out by means of the program CPPTRAJ^87^ in Amber 20.

In order to unveil energetic basis for conformational changes of M-RAS, FELs were built by using root-mean-square deviations (RMSDs) of non-hydrogen atoms in M-RAS and the distance of residues D43 away from E73 as the reaction coordinates recorded in the SGT with the following the equation^88^^,^^89^

(9) $$G_{i}=-k_{B}Tln(\frac{N_{i}}{N_{\max}})$$

from which ${\;k}_{B}$ is Boltzmann’s constant, *T* is the temperature of simulation systems and 300 K is set in this work. The parameter $N_{i}$ is the population of the *i*th bin and $N_{\max}$ is the population of the most populated bin. Bins without population are set as an artificial barrier that is scaled as the lowest probability. The construction of FELs was achieved with the program PyReweighting developed by Miao et al.^78^.

## Results and discussion

### Structural flexibility and internal dynamics of M-RAS

**Figure 2.**
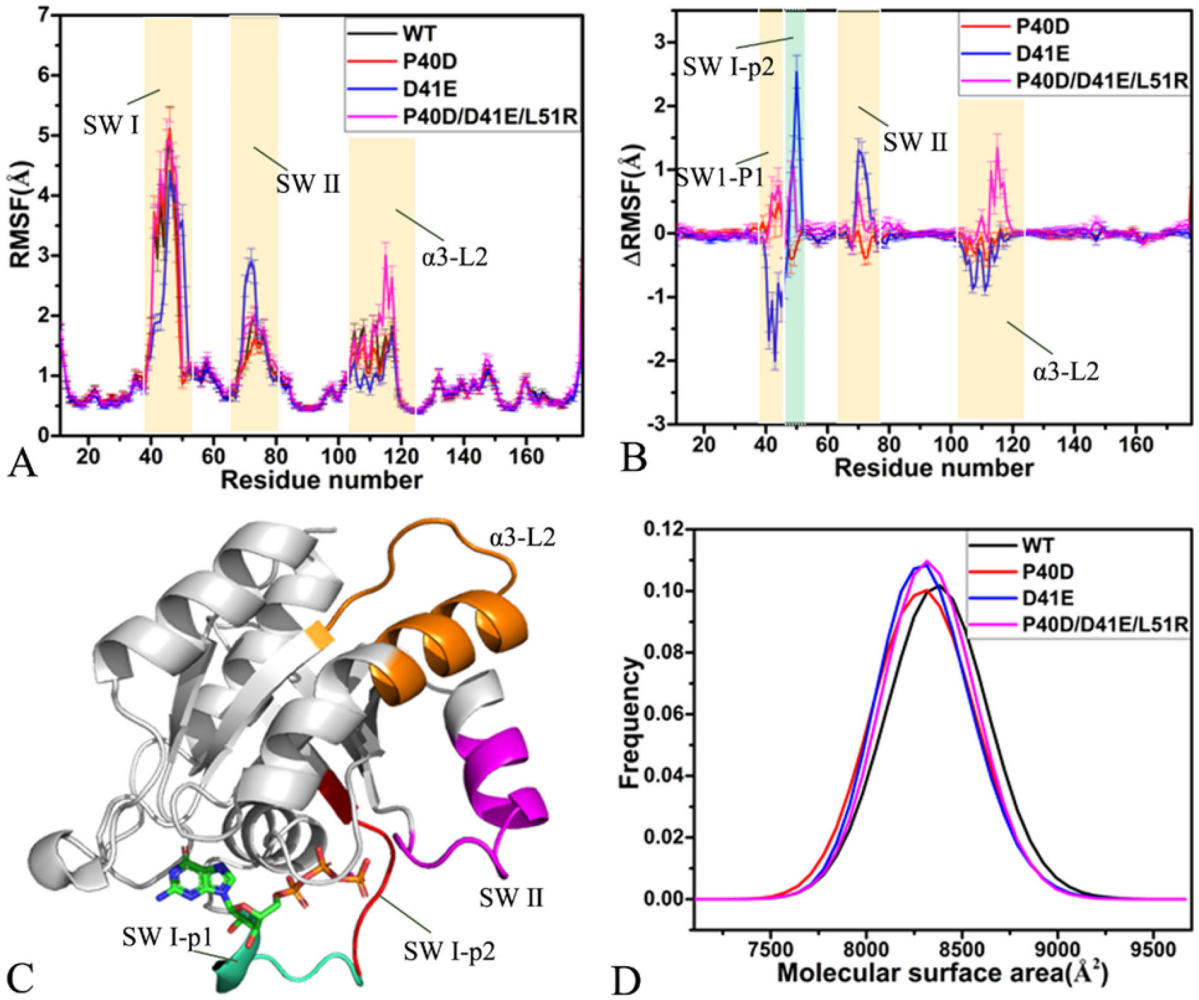
Structural flexibility and molecular surface areas of M-RAS in four systems: (A) RMSFs of the C_α_ atoms from M-RAS, (B) difference in RMSFs between the WT and mutated M-RAS, (C) structural domains with obvious alterations of RMSFs and (D) molecular surface area of the WT and mutated M-RAS.

The time course of root-mean-square deviations (RMSDs) for non-hydrogen atoms in M-RAS was calculated by referencing the initial minimised structures to access structural fluctuations of four simulated systems (Figure S1). It is found that the structures of M-RAS in four systems fluctuate at a common range from 1.35 to 3.73 Å, implying that mutations do not generate a large effect on the total structural fluctuations of M-RAS. The RMSFs of the C_α_ atoms from M-RAS were computed on the basis of the SGT to evaluate the flexible extent of the structural domains in M-RAS (Figure 2(A)). Meanwhile, the difference in the RMSFs between the GTP-bound mutated M-RAS and the GTP-bound WT one was also calculated according to the equation $\Delta \mathrm{RMSF}={\mathrm{RMSF}}_{\mathrm{mutant}}-{\mathrm{RMSF}}_{WT},$ of which ΔRMSF, ${\mathrm{RMSF}}_{\mathrm{mutant}}$ and ${\mathrm{RMSF}}_{WT}$ signify the difference of the RMSFs, the RMSF of the mutated M-RAS, and that of the WT one (Figure 2(B)). The structural domains corresponding to evident changes in the RMSFs were depicted in Figure 2(C). As observed in Figure 2(A), the switch domains SW I and SW II together with the domain α3-L2 show high flexibility, in particular for the SW I (Figure 2(A,C)). Based on Figure 2(B), D41E evidently decreases the RMSF of part 1 (SW I-p1) of the switch domain SW I while P40D and P40D/D41E/L51R increase the RMSF of this region (Figure 2(B,C)), suggesting that D41E obviously weakens the structural flexibility of the SW I-p1 but P40D and P40D/D41E/L51R strengthen the structural flexibility of this region. Mutations D41E and P40D/D41E/L51R increase the RMSF of part 2 (SW I-p2) of the SW I but P40D reduces the RMSF of this region (Figure 2(B,C)), indicating that D41E and P40D/D41E/L51R enhance the structural flexibility of the SW I-p2 while P40D slightly abates the structural flexibility. As shown in Figure 2(B), D41E and P40D/D41E/L51R strengthen the flexibility of the switch domain SW II while P40D only softly weakens that of this region. D41E evidently abates the structural flexibility of the domain α3-L2 but P40D/D41E/L51R greatly enhances that of this region, differently P40D only slightly weakens the structural flexibility of the α3-L2 (Figure 2(B,C)). The ^31^P NMR spectra and crystal structures of the GppNHp-bound mutated M-RAS determined by Shima et al. suggested that the switch domains of M-RAS have high structural flexibility^19^.

The solvent-accessible surface area (SASA) of receptors is usually described using their molecular surface area (MSA). To monitor influences of P40D, D41E, and P40D/D41E/L51R on SASA of M-RAS, MSAs of the GTP-bound WT and mutated M-RAS were calculated using the linear combination of pairwise overlap (LCPO) method^90^ (Figure 2(D)). As observed in Figure 2(D), mutations P40D, D41E, and P40D/D41E/L51R lead to a decrease of 67.5 Å2 in MSAs, implying that these three mutations all reduce the contacts of M-RAS with solvents and affect conformational changes of M-RAS.

To evaluate the effect of P40D, D41E, and P40D/D41E/L51R on structural compact extents of M-RAS, radius of gyrations (Rgs) of M-RAS were computed based on the SGT, and their frequency distributions were provided at supporting information (Figure S2). The Rg of the GTP-bound P40D M-RAS is decreased by 0.1 Å relative to that of the GTP-bound WT M-RAS while the Rg of the GTP-bound D41E M-RAS is increased by 0.15 Å, indicating that P40D strengthens the structural compact extent of M-RAS but D41E reduces that of M-RAS compared to the WT M-RAS (Figure S2). Although the peak values for the Rgs of the GTP-bound WT and P40D/D41E/L51R M-RAS are distributed at the same position of 15.58 Å, the distributed shape for the Rg of the GTP-bound P40D/D41E/L51R M-RAS totally moves towards the right relative to that of the GTP-bound WT M-RAS (Figure S1), thus P40D/D41E/L51R slightly decreases the structural compact extent of M-RAS.

DCCMs were estimated to understand the impacts of P40D, D41E, and P40D/D41E/L51R on the internal dynamics of M-RAS (Figure S3). The extents of the PCMs and ACMs between residues of M-RAS were respectively scaled in red (or yellow) and dark blue (or blue), which is embodied by the colour bar. In the GTP-bound WT M-RAS, the region R1 describes the strong ACM of the switch domain SW I (residues 38–51) relative to the P-loop (16–28), and the region 2 characterises the strong ACM of the switch domain SW II (residues 67–85) relative to SW I (Figure S3(A)). Meanwhile for the GTP-bound WT M-RAS, the region R3 does not generate the obvious ACM between residues 90–110 and the SW II while the region R4 embodies the slight ACM between the loop L2 (residue 110–120) and the SW I (Figure S3(A)). By referencing the WT M-RAS, P40D, and P40D/D41E/L51R slightly strengthen the ACM of the SW II relative to the SW I indicated by the region R2 (Figure S3(B,D)) but D41E leads to the disappearance of the ACM between the SW II and the SW I (Figure S3(C)). Compared to the WT M-RAS, P40D hardly influences the ACM of the SW I relative to the P-loop represented by the region R1 (Figure S3(B)) but D41E and P40D/D41E/L51R greatly weaken the ACM between the SW I and the P-loop (Figure S3(C,D). Although the ACM does not appear between residues 90–110 and the SW II in the GTP-bound WT and P40D M-RAS (Figure S3(A,B)), the strong ACM between residues 90–110 and the SW II is observed in the GTP-bound D41E and P40D/D41E/L61R M-RAS. By comparison with the GTP-bound WT, P40D, and D41E M-RAS, P40D/D41E/L51R obviously strengthen the ACM of the loop L2 relative to the SW I reflected by the region R4 (Figure S3(D)). It is obtained from the above analyses that the regions relating to obvious changes in the internal dynamics of M-RAS are mainly involved in the switch domains SW I and SW II, which is in good agreement with the RMSF results.

**Figure 3.**
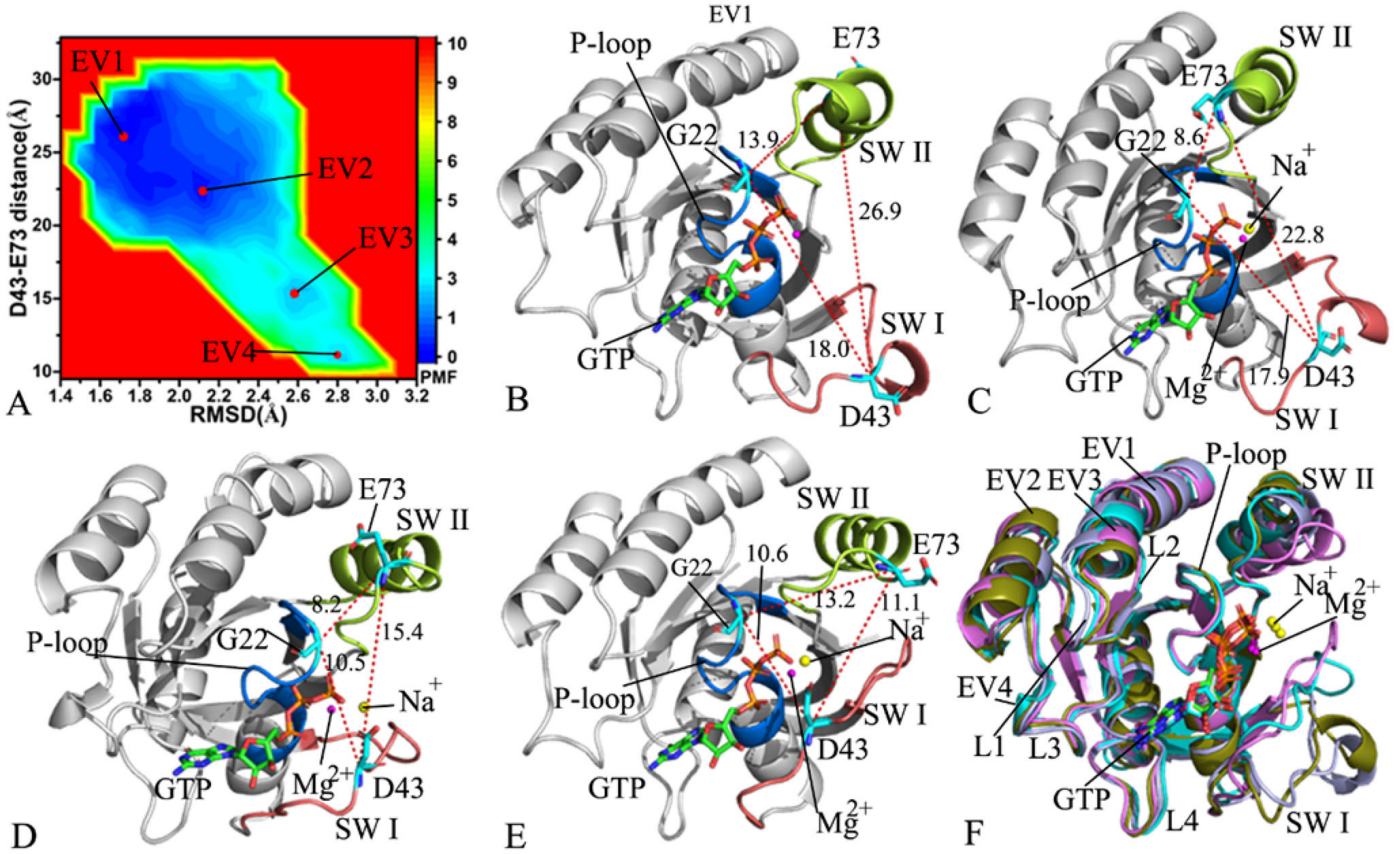
FEL and representative structures of the GTP/WT M-RAS complex: (A) FEL with four energy valleys EV1-EV4, (B) the structure located at the EV1, (C) the structure situated at the EV2, (D) the structure falling into the EV3, (E) the structure trapped in the EV4 and (F) structural superimposition of the GTP/WT M-RAS complexes located at the EV1-EV4. In this figure, M-RAS, GTP and ions Mg^2+^ and Na^+^ were displayed in cartoon, stick and ball patterns. The PMF was scaled in kcal/mol and the distance was indicated in Å.

In summary, mutations P40D, D41E, and P40D/D41E/L51R not only change the structural compact extent of M-RAS and contact extent of M-RAS with solvents but also highly affect the structural flexibility and dynamics behaviour of the switch domains. Multiple experimental works indicated that ligand binding and mutations in different sites of RAS proteins also exert a significant effect on the structural flexibility of the switch domains^12^^,^^91–94^, which possibly alters the activity of RAS proteins, including M-RAS. The previous reports verified that high flexibility is a main feature of the switch regions of RAS proteins, which enables conformational transformation associated with a GDP/GTP exchange^19^^,^^24^^,^^95^. Thus, the changes in structural flexibility and internal dynamics of M-RAS disturb the binding of M-RAS to its effectors.

### Free energy profiles relating with conformational transition of M-RAS

**Figure 4.**
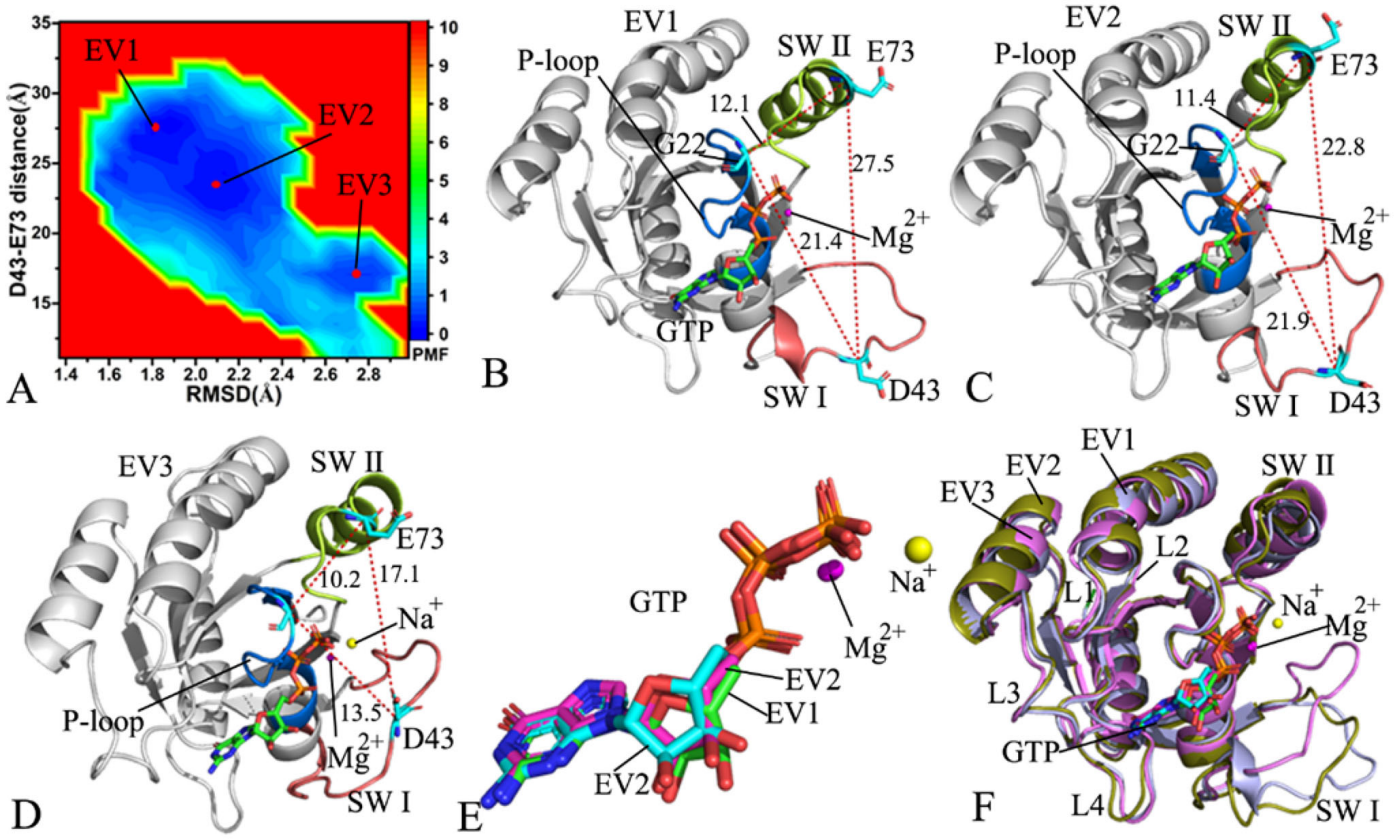
FEL and representative structures of the GTP/P40D M-RAS complex: (A) FEL with three energy valleys EV1-EV3, (B) the structure located at the EV1, (C) the structure situated at the EV2, (D) the structure falling into the EV3, (E) structural superimposition of GTP and magnesium ions trapped at the EV1-EV3 and (F) structural alignment of the GTP/P40D M-RAS complexes situated at the EV1-EV3. In this figure, M-RAS, GTP and ions Mg^2+^ and Na^+^ displayed in cartoon, stick and ball patterns. The PMF was measured in kcal/mol and the distance was represented in Å.

**Figure 5.**
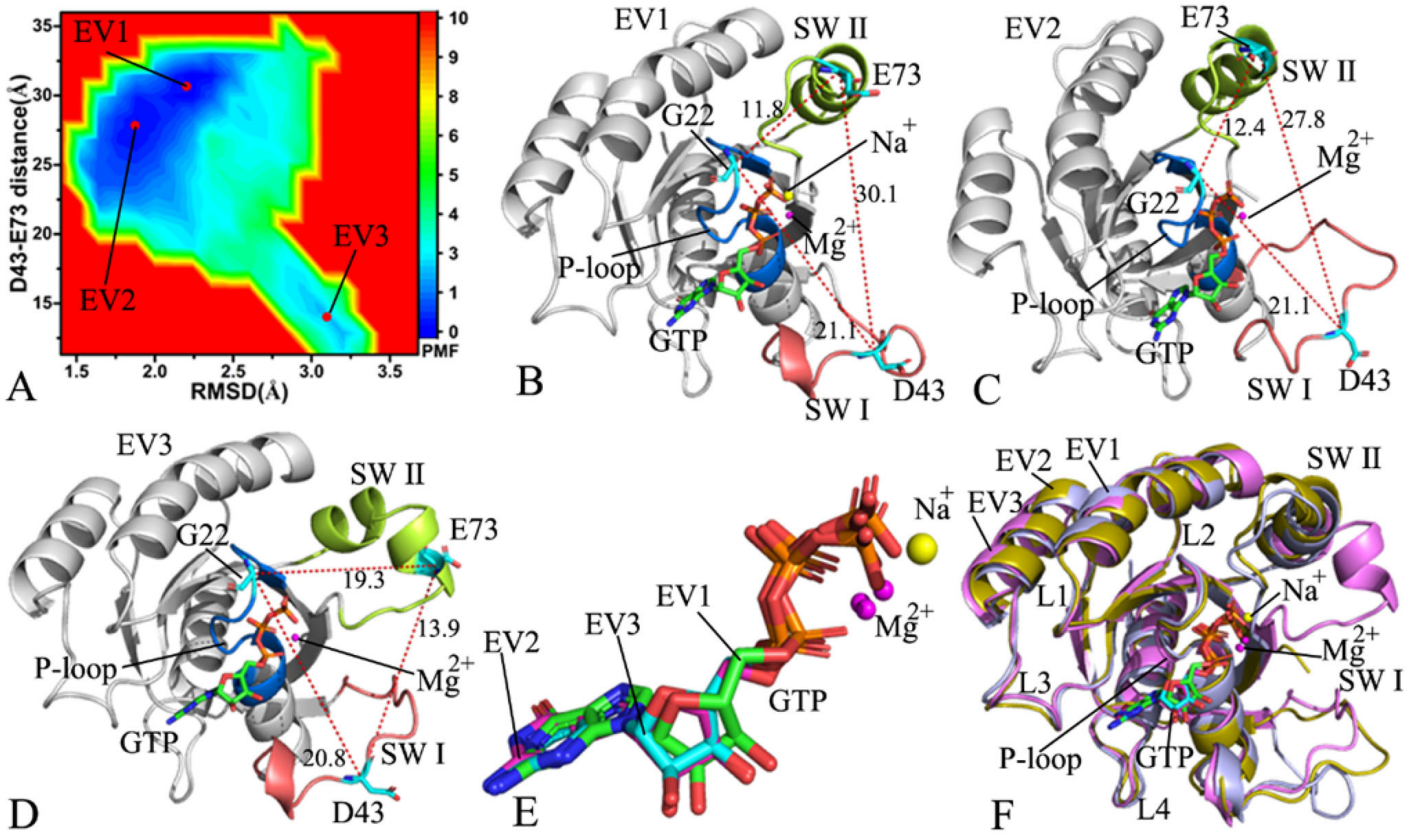
FEL and representative structures of the GTP/D41E M-RAS complex: (A) FEL with three energy valleys EV1-EV3, (B) the structure situated at the EV1, (C) the structure trapped at the EV2, (D) the structure corresponding to the EV3, (E) structural superimposition of GTP and magnesium ions falling into the EV1-EV3 and (F) structural alignment of the GTP/D41E M-RAS complexes situated at the EV1-EV3. In this figure, M-RAS, GTP and ions Mg^2+^ and Na^+^ depicted in cartoon, stick and ball patterns. The PMF was measured in kcal/mol and the distance was represented in Å.

**Figure 6.**
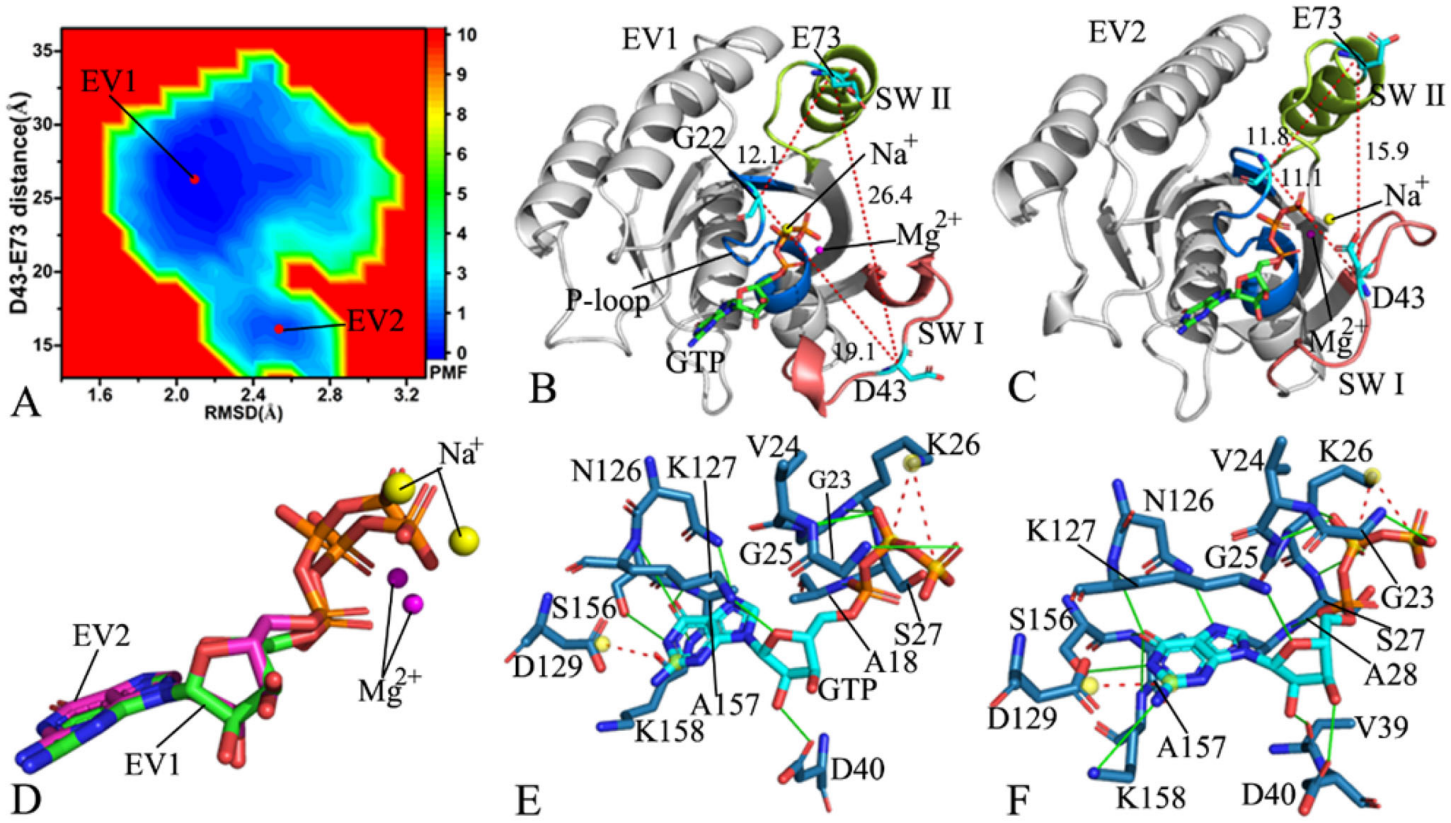
FEL and representative structures of the GTP-P40D/D41E/L51R M-RAS complex: (A) FEL with two energy valleys EV1 and EV2, (B) the structure located at the EV1, (C) the structure trapped at the EV2, (D) structural superimposition of GTP and magnesium ions situated at the EV1-EV2 and (E) interaction network of GTP with M-RAS in the incompact state of the switch domains and (F) interaction network of GTP with M-RAS in the compact state of the switch domains. In this figure, M-RAS, GTP and ions Mg^2+^ and Na^+^ depicted in cartoon, stick and ball patterns. The PMF was scaled in kcal/mol and the distance was indicated in Å.

FELs are usually utilised to characterise the thermodynamics and kinetics of a ligand-target and protein-solvent systems at certain conditions^96^. To unveil the energetic basis with regard to the conformational transition of M-RAS, FELs were built by using the RMSDs of non-hydrogen atoms from M-RAS and the distance between the C_α_ atoms of residues D43 and E73 as reaction coordinates (RCs). The RMSDs of non-hydrogen atoms from M-RAS can efficiently embody the total structural fluctuation of M-RAS while the distance of the C_α_ atom of D43 in the SW I away from that of E73 in the SW II of M-RAS can rationally characterise the state transformation of the switch domains, which explains the reason why we choose them as RCs. The constructed FELs and the representative structural information were depicted in Figure (3–6).

With regard to the GTP/WT M-RAS complex, M-RAS comes across four different conformational subspaces situated at the energy valleys EV1-EV4 through the entire GaMD simulations (Figure 3(A)). The distances of D43 in the SW I away from E73 in the SW II are 26.9, 22.8, 15.4, and 11.1 Å in the structures EV1, EV2, EV3, EV4 (Figure 3(B–E)), respectively, showing that the switch domains in the structure EV1 are the most incompact and that in the structure EV4 the most compact. The depth of the EV1 is much deeper than that of the EV4 on the basis of colour bar, thus the conformational transition from EV1 to EV4 is more difficult than that of EV4 to EV1. As a result, the conformations of the GTP-bound WT M-RAS are mostly populated at the most incompact state of the switch domains. The structures of GTP and magnesium ions (Mg^2+^) trapped at the EV1-EV4 were superimposed together to evaluate the stability of GTP and Mg^2+^ (Figure S4(A)). Except for the slight deviation of the phosphate group in GTP, the other parts of GTP and magnesium ions are aligned well, suggesting that GTP and magnesium ions are stable during GaMD simulations. At the same time, M-RASs located at the EV1-EV4 were also superimposed together to access the conformational stability of M-RAS (Figure 3(F)). The results verify that the switch domains SW I and SW II are located at the highly disordered states, moreover, the disordered extent of SW I is higher than that of SW II. The SW I in the most compact state of the switch domains goes closely to the GTP while that in the most incompact state of the switch domains leaves the GTP. Therefore the disordered state of the SW I affects interactions of GTP with the SW I. To check this issue, the interaction network of GTP with M-RAS was analysed on the most incompact and compact states of the switch domains by using protein − ligand interaction profiler (PLIP) server^97^^,^^98^ and the results was depicted Figure S4(B,C). In two states, GTP generates hydrogen bonding interactions (HBIs) with common residues G23, V24, G25, K26, S27, A28, N126, K127, D129, A157 and A158 and three salt bridge interactions with K26 and D129. The disordered state of the SW I leads to the disappearance of interactions between GTP and two residues D41 and Y42 in the SW I in the most incompact state of the switch domains (Figure S4(B,C)), meanwhile, the interaction of GTP with residue S156 also misses in the most incompact state of the switch domains. It is well known that the SW I is involved in the binding of M-RAS to its effectors, hence the high disordered state of the SW I certainly affects the binding of M-RAS to effectors and disturbs the activity of M-RAS. In addition, a sodium ion (Na^+^) appears at the EV2-EV4, which may provide compensation for the change of polar environment caused by conformational alterations of the switch domains.

For the GTP/P40D M-RAS complex, the conformations of M-RAS are mainly populated at three energy valleys EV1-EV3 during GaMD simulations (Figure 4(A)). The distances of D43 away from E73 in the structures EV1, EV2 and EV3 are 27.5, 22.8 and 17.1 Å, respectively (Figure 4(B–D)), suggesting that the switch domains of the GTP-bound P40D M-RAS are most tightly packed in the structure EV1 while the switch domains in the structure EV3 state most loosely packed. The structures of GTP and Mg^2+^ situated at the EV1-EV3 were aligned together to check the stability of these two ligands (Figure 4(E)). The results indicate that GTP and Mg^2+^ agree well with each other in three energetic states, implying that these two ligands are stable through the entire GaMD simulations. To examine the structural stability of M-RAS, the M-RASs falling into the EV1-EV3 were superimposed together (Figure 4(F)). It is found that the SW I shows a highly disordered state while the SW II only yields a slight deviation. The SW I of the structure EV1 goes away from GTP while that of the structure EV3 goes closely to GTP, which generates influences on the binding of GTP to M-RAS. To clarify this issue, the interaction network between GTP and M-RAS was analysed with the PLIP sever and the results were exhibited at Figure S5. The only difference is that the HBI of GTP with D40 in the SW I of the structure EV3 loses at the structure EV1. Compared to the WT M-RAS, the depth of the EV1-EV3 are almost the same in the P40D mutated M-RAS, thus the conformations of the P40D mutated M-RAS are almost averagely populated at the EV1-EV3, implying that P40D alters the conformation distribution of M-RAS. By referencing to the most compact state of the GTP/WT M-RAS complex, P40D leads to the disappearance of two HBIs between GTP and residues D41 and Y42 but directly induces a new HBI of D40 with GTP in the GTP/P40D M-RAS complex. Additionally, a sodium ion (Na^+^) appears at the structure EV3, implying an electrostatic compensation on polarity alterations due to P40D.

On the GTP/D41E M-RAS complex, M-RAS goes across three main conformational subspaces distributed at three energy valleys EV1-EV3 (Figure 5(A)). The distances of D43 away from E73 in the structures EV1-EV3 are 30.1, 27.8 and 13.9 Å, respectively (Figure 5(B–D)), signifying that the switch domains of the EV1 have the loosest state while the switch domains of the EV3 have the tightest state. The depth of the EV3 is shallower than that of the EV1 and EV2 according to colour bar (Figure 5(A)), hence the transition probability from the EV3 to the EV1 and EV2 is higher than that form the EV1 and EV2 to the EV3. As a result, the switch domains of the GTP-bound D41E M-RAS mainly exist in a loose state. Based on the colour bar in Figure 3(A) and 5(A), the energy barrier between the most incompact and compact states of the switch domains in the WT M-RAS is about 1.0 kcal/mol lower than that in the D41E M-RAS, which enhances the difficulty of the conformation transition from the loose switch domains to the tight ones. The structures of GTP and Mg^2+^ located at the EV1-EV3 were superimposed together to access the stability of these two ligands in the binding pocket (Figure 5(E)). Apart from a slight deviation of Mg^2+^, GTP is aligned well, showing that GTP and Mg^2+^ are stable through GaMD simulations. The structures of M-RAS trapped at the EV1-EV3 were superimposed to probe the structural stability of M-RAS (Figure 5(F)). It is observed that the SW I and SW II are highly disordered in the D41E mutated state of M-RAS. The SW I in the structure EV1 goes away from GTP while that in the structure EV3 comes close to GTP, which certainly produces important impacts on the binding of GTP to M-RAS. The helix from the SW II of the EV3 is divided into two parts and one part goes way from the phosphate group of GTP, which also affects the binding of GTP to M-RAS. To understand this issue, the structures of the loosest and tightest switch domains were used to analyse the interaction network of GTP with the D41E M-RAS (Figure S6). Apart from common interactions of GTP with conserved residues G23, V24, G25, K26, S27, A28, D129, N126, S156, A157, and K158, the HBIs of the phosphate group from GTP with G70 and Q71 in the SW II are identified in the loosest state of the switch domains (Figure S6(A)), but a π–π interaction of the guanine group from GTP with the phenyl group of Y42 from the SW I is detected in the tightest state of the switch domains (Figure S6(B)). Compared to the WT M-RAS, D41E generates more obvious effect on the interactions of GTP with the switch domains. Besides, a sodium ion (Na^+^) is found at the structure EV1, which reflects a change of polar environment because of D41E.

As for the GTP-bound P40D/D41E/L51R M-RAS, M-RAS comes through two main conformational subspaces located at the energy valleys EV1 and EV2 (Figure 6(A)). The distances of D43 away from E73 in the structures EV1 and EV2 are 26.4 and 15.9 Å (Figures 6(B,C)), separately, suggesting that the compact extent of the switch domains in the structure EV1 is smaller than that in the structure EV2. By comparison with the WT M-RAS, P40D/D41E/L51R reduces the energy barrier between the loose and tight states of the switch domains, which makes the reciprocal conformation transition between two states easier, thus more conformations are populated at the tight state of the switch domains in the P40D/D41E/L51R M-RAS than that in the WT M-RAS (Figures 3A and 6A). The structures of GTP and Mg^2+^ in the structures EV1 and EV2 were superimposed to study the stability of these ligands (Figure 6(D)). Although the guanine group and the middle ring of GTP are aligned well, the phosphate group of GTP and Mg^2+^ produce evident deviation. Meanwhile, the structures of M-RAS in the structures EV1 and EV2 were also superimposed to understand the structural stability of M-RAS (Figure S7). The SW I in EV1 leaves the GTP and that in EV 2 goes closely to GTP, which affects the binding of GTP to M-RAS. The interaction network of GTP with M-RAS in the structures EV1 and EV2 were analysed with the PLIP sever (Figure 6(E,F)). By reference to the structure EV1, a new HBI between GTP and V39 in the SW I appears at the structure EV2. Additionally, a sodium Na + is detected in the structure EV1 and EV2, which possibly provides compensation for the changes in polarity caused by conformational alterations of M-RAS because of P40D/D41E/L51R.

**Figure 7.**
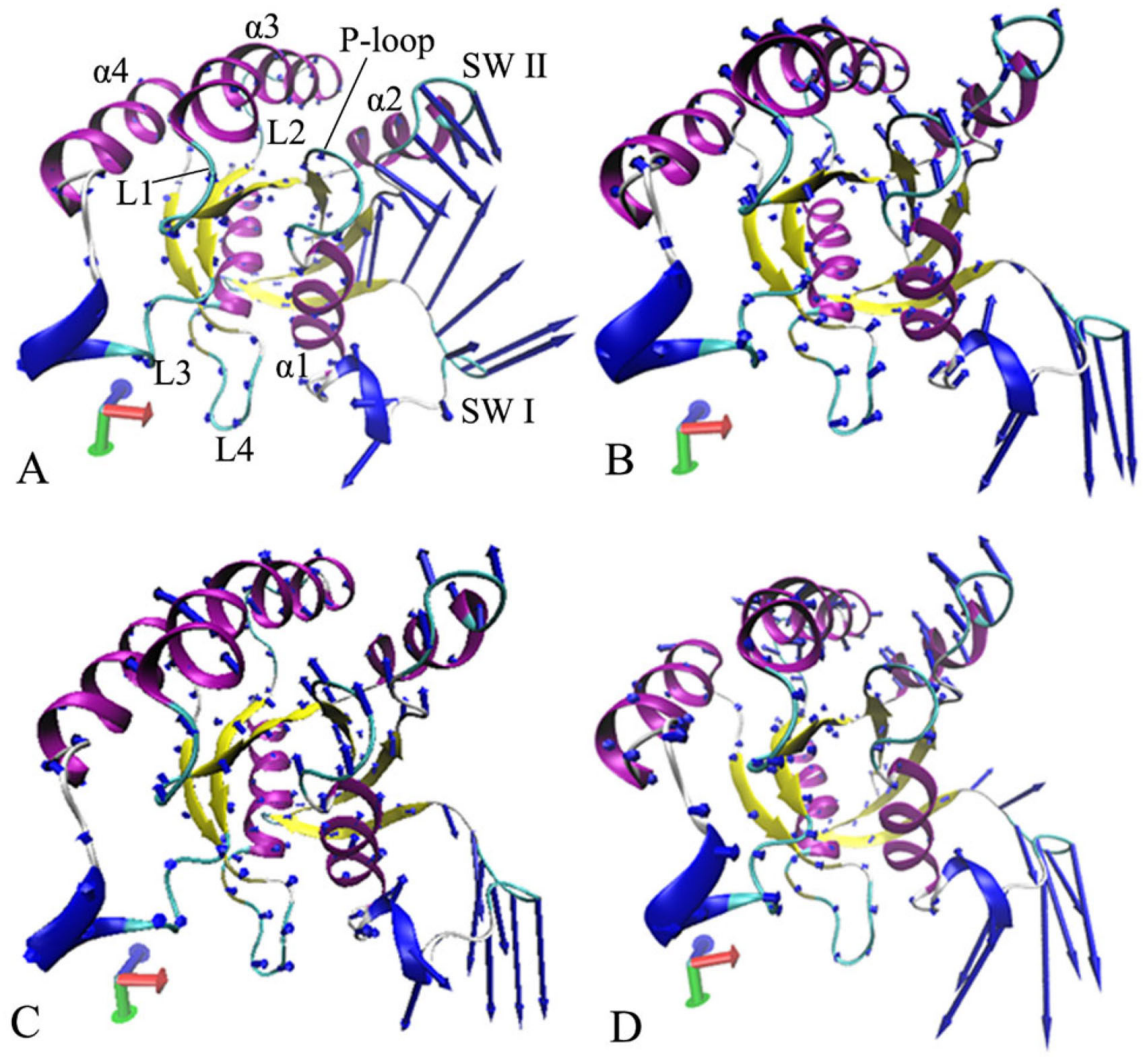
Collective motions of the structure domains from the GTP-bound WT and mutated M-RAS: (A) the GTP-bound WT M-RAS, (B) the GTP-bound P40D M-RAS, (C) the GTP-bound D41E M-RAS and (D) the GTP-bound P40D/D41E/L51R M-RAS.

On the basis of the above analyses, three interesting findings are obtained: (1) P40D, D41E and P40D/D41E/L51R all induce less energetic states and change population at different states, implying conformation rearrangement of M-RAS, (2) a different number of sodium ions appear at different energetic states, implying an alteration of polarity near the phosphate group and (3) the relative position and orientation of the SW I relative to GTP change due to mutations, which affects binding of GTP to M-RAS, meanwhile the disordered state of the SW II also impacts binding of GTP to M-RAS. More interestingly, the SW I mostly overlaps with the binding interface of M-RAS to its effectors, the highly disordered state of the SW I can result in instable contacts with binding regions of M-RAS to effectors, thus mutations produce a significant effect on the activity of M-RAS. The work of Ye et al. indicated that the predominance of state 1 of the switch domains in M-RAS is likely involved in its weak binding ability to the RAS effectors and implies importance of the tertiary structure factor in small GTPase-effector interaction^27^. The conformational changes accompanying the state transition imply a common mechanistic basis inherent in the high flexibility of the switch regions^19^. The previous works of other RAS proteins verified that the conformational alterations of the switch domains affect the binding of RAS proteins to effectors^29^^,^^99–103^.

### Collective motions of M-RAS revealed by PCA

PCA is usually utilised to comprehend and analyse the dynamic data recorded in the cAAMD trajectories. For this work, PCA was performed on the SGT of the GTP-bound WT and mutated M-RAS to probe the overall dynamics of M-RAS by using the program CPPTRAJ in Amber 20. PCA can obtain a diagonal covariance matrix with eigenvectors and eigenvalues, in which eigenvectors describe concerted motions of M-RAS and eigenvalues characterise overall structure fluctuations of M-RAS along an eigenvector. The function of eigenvalues as eigenvectors was displayed in Figure S8. The first five eigenvectors account for 91.5, 92.7, 85.2, and 88.9% of the total movements of the GTP-bound WT, P40D, D41E, and P40D/D41E/L51R M-RAS, separately. By referencing the WT M-RAS, the first eigenvalue of the P40D M-RAS is increased because of P40D and the first eigenvalues of the D41E and P40D/D41E/L51R M-RAS are decreased due to these two mutations, implying that P40D strengthens the structural fluctuations of M-RAS along the first eigenvector but D41E and P40D/D41E/L51R weaken the structural fluctuation of M-RAS.

**Figure 8.**
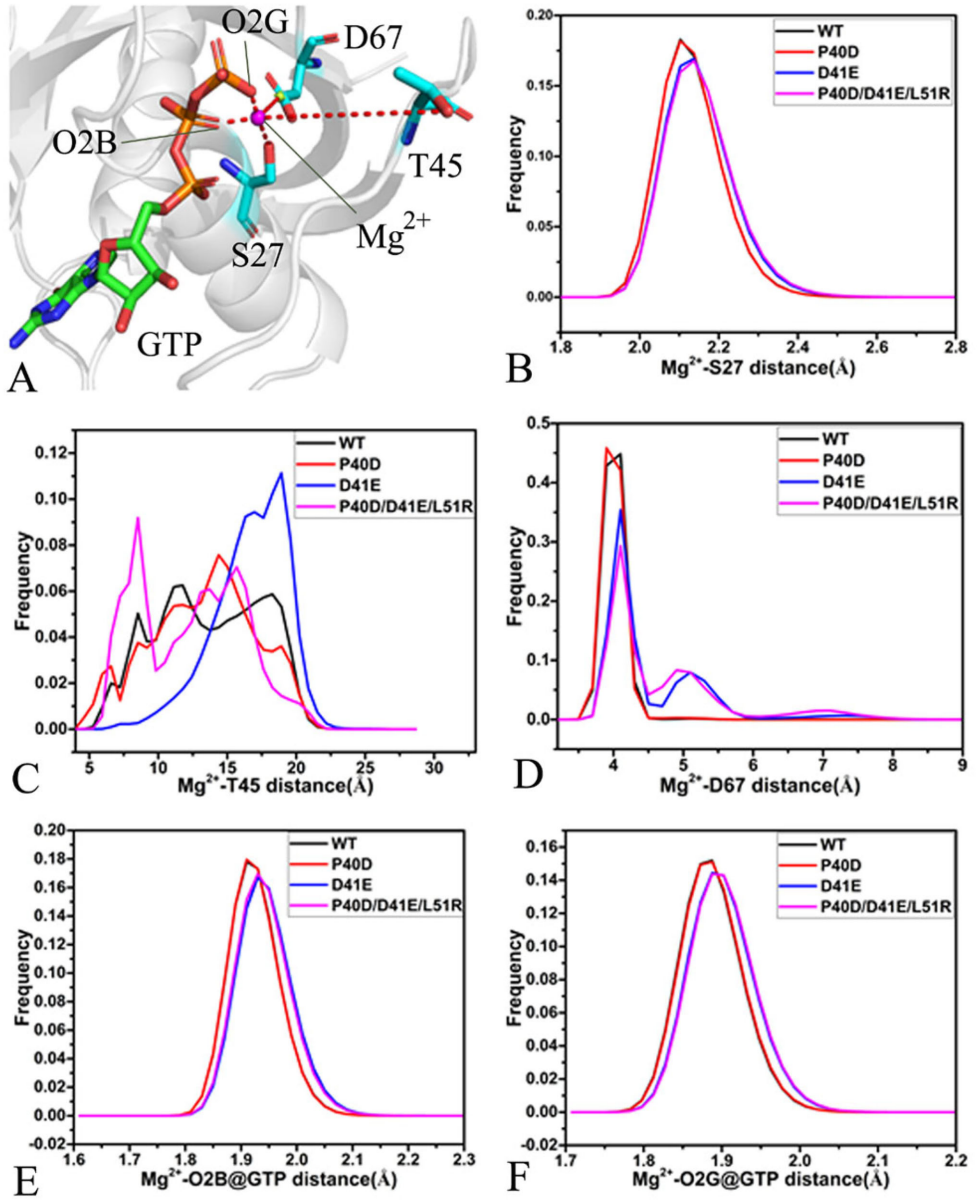
Interactions of magnesium ion with GTP, P-loop, SW I and SW II: (A) geometric information involved in interactions of magnesium ion, (B) the frequency distribution for the distance of Mg^2+^ away from the oxygen atom OG of S27, (C) the frequency distribution for the distance between Mg^2+^ and oxygen atom O of T45, (D) the frequency distribution for the distance of Mg^2+^ away from the mass centre of the oxygen atoms OD1 and OD2 of D67, (E) the frequency distribution for the frequency distribution forthe distance between Mg^2+^ and the oxygen atom O2B of GTP and (F) the frequency distribution for the distance of Mg^2+^ away from the oxygen atom O2G of GTP.

To explore the changes in concerted motions of M-RAS caused by three mutations, the first eigenvector was visualised by means of the program VMD^104^ (Figure 7). Two interesting findings were observed: (1) the switch domains show strong collective motions and mutations impact the fluctuation amplitude of the switch domains, which agrees with the previous RMSF analyses and (2) mutations induce the changes in fluctuation tendency of the switch domains along the first eigenvector. For the GTP/WT M-RAS complex, the SW I and SW II have a strong and opposite collective motions and form a tendency to go closely to each other (Figure 7(A)). Compared to the WT M-RAS, three mutations not only change the tendency of concerted movements of the SW I and SW II but also abate the structural fluctuation amplitude of the switch domain SW II (Figure 7(B–D)), thus mutations alter dynamics behaviour of M-RAS.

A magnesium ion is located at the pocket encircled by the P-loop, SW I, and SW II and near the phosphate group of GTP. The distances of Mg^2+^ away from the oxygen atom OG of S27 in the P-loop, the oxygen atom O of T45 in the SW I, the mass centre of oxygen atoms OD1 and OD2 from D67 in the SW II and the oxygen atoms O2B and O2G of GTP were calculated to probe the reason relating with the conformational changes. The position geometry and the frequency distribution of the distances were depicted in Figure 8 and their time evolution was displayed Figure S9. The distance of Mg^2+^ away from the oxygen atom OG of S27 in the SW I fluctuates from 1.81 to 3.23 Å and it is stable through the entire GaMD simulations (Figure 8(A) and S9(A)). The distances between Mg^2+^ and the OG of S27 of the GTP-bound WT and P40D M-RAS are distributed at 2.10 Å while the ones of the GTP-bound D41E and P40D/D41E/L51R M-RAS are located at 2.15 Å, thus D41E and P40D/D41E/L51R slightly weaken the interaction of Mg^2+^ with S27 relative to the WT M-RAS but P40D hardly impacts this interaction (Figure 8(B)). The distance of Mg^2+^ away from the oxygen atom O of T45 in the SW I fluctuates in a range from 4.12 to 27.45 Å (Figure 8(A) and S9(B)), which not only implies that the relative position of Mg^2+^ to T45 is unstable during GaMD simulations but also indicates that the SW I highly sways near Mg^2+^. It is observed from Figure 8(C) that the distance between Mg^2+^ and the O of T45 in the WT, P40D and P40D/D41E/L51R M-RAS is populated at multiple peak values while that in the D41E M-RAS is distributed at two peak values (Figure 8C), which further supports that this distance is unstable. This result provides rational explanation for the high disorder of the SW I. As shown in Figure S9(C), the fluctuation range of the distance between Mg^2+^ and the mass centre of the oxygen atoms OD1 and OD2 of D67 in the SW II is 3.52–10.59 Å, suggesting that this distance is also instable through the entire GaMD simulations. This distance in the P40D M-RAS is distributed at 3.9 Å while that in the WT M-RAS is located at 4.1 Å (Figure 8(D)), which rationally explains the reason of the lower disorder of the SW II in the P40D M-RAS than the WT one. The frequency of the distance between Mg^2+^ and the mass centre of OD1 and OD2 of D67 are situated at the peak values of 4.1, 5.1 and 7.1 Å in the D41E and P40D/D41E/L51R M-RAS (Figure 8(D)), verifying that D41E and P40D/D41E/L51R weaken the interaction of Mg^2+^ with D67 compared to the WT M-RAS. The distances of Mg^2+^ away from the oxygen atoms O2B and O2G of GTP are separately situated at the range of 1.74–2.33 and 1.73–2.21 Å (Figure 8(A), S^9^(D) and S^9^(E)), indicating the relative position of Mg^2+^ to the phosphate group of GTP is highly stable during GaMD simulations. The frequency shape of these two distances in the D41E and P40D/D41E/L51R M-RAS move towards the right (Figure 8(E, 8F)), implying that D41E and P40D/D41E/L51R slightly weaken the interactions of Mg^2+^ with the O2B and O2G of GTP relative to the WT and P40D M-RAS.

**Figure 9.**
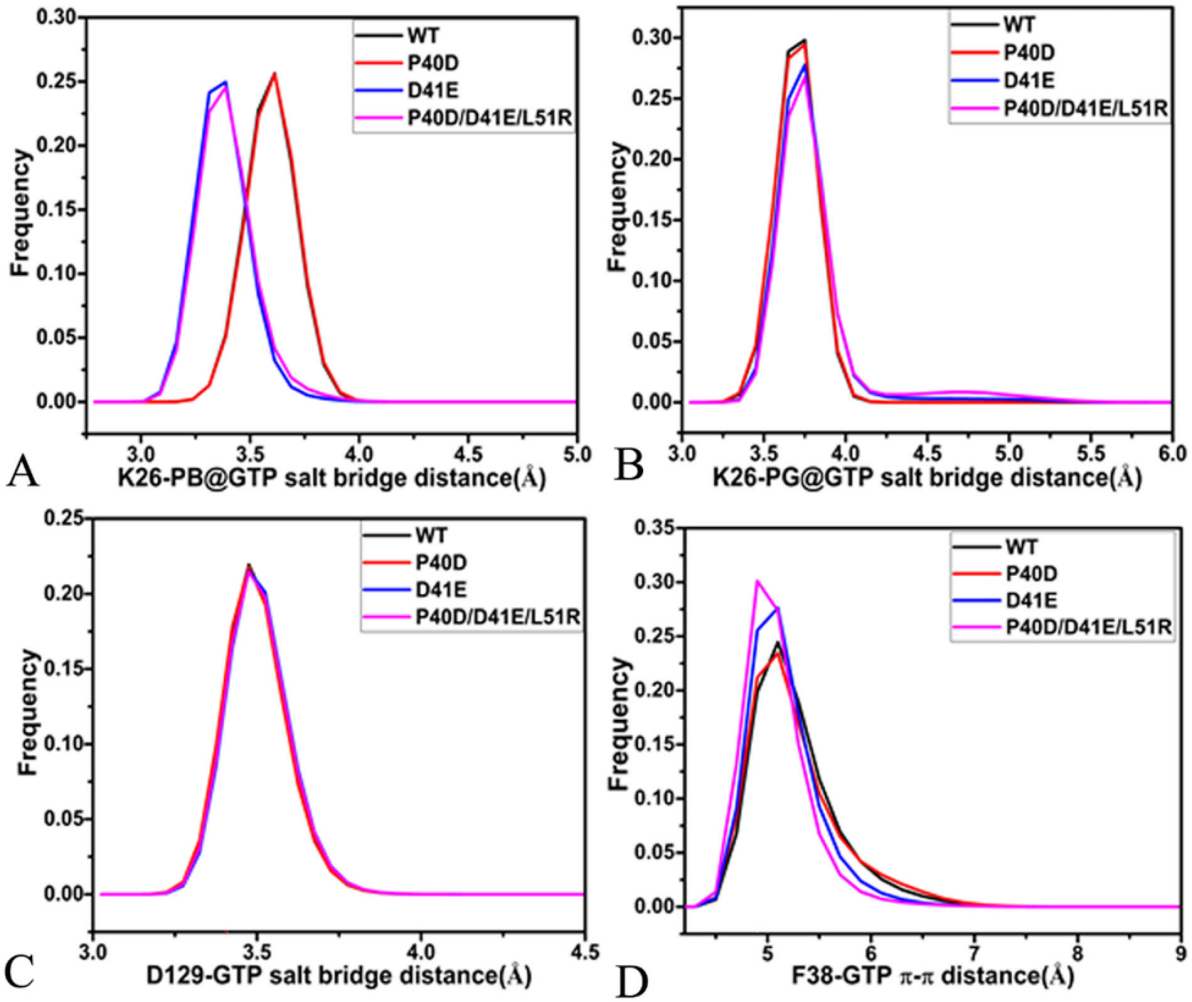
The salt bridge interactions and the π–π interaction of GTP with M-RAS: (A) the frequency distribution of the distance between NZ and PB for the salt bridge interaction of GTP with K26, (D) the frequency distribution of the distance between NZ and PG for the salt bridge interaction of GTP with K26, (C) the frequency distribution of the distance for the salt bridge between D129 and GTP and (D) the frequency distribution of the distance for the π-π interaction of F28 with GTP.

On the basis of the aforementioned analyses, three mutations not only change the direction of collective motions of the switch domains but also inhibit the fluctuation amplitudes of the SW II by comparison with the WT M-RAS. The instability in the relative positions of Mg^2+^ to the SW I and SW II drives the high disorder of the switch domains, which implies that Mg^2+^ plays an important role in in the GTP/M-RAS binding^105^. The switch domains of M-RAS are involved in interactions with nucleotides and binding of M-RAS to effectors^19^^,^^27^. Thus, the conformational alterations of the switch domains caused by P40D, D41E and P40D/D41E/L51R affect the activity of M-RAS.

### Analyses of interaction network between GTP and M-RAS

**Figure 10.**
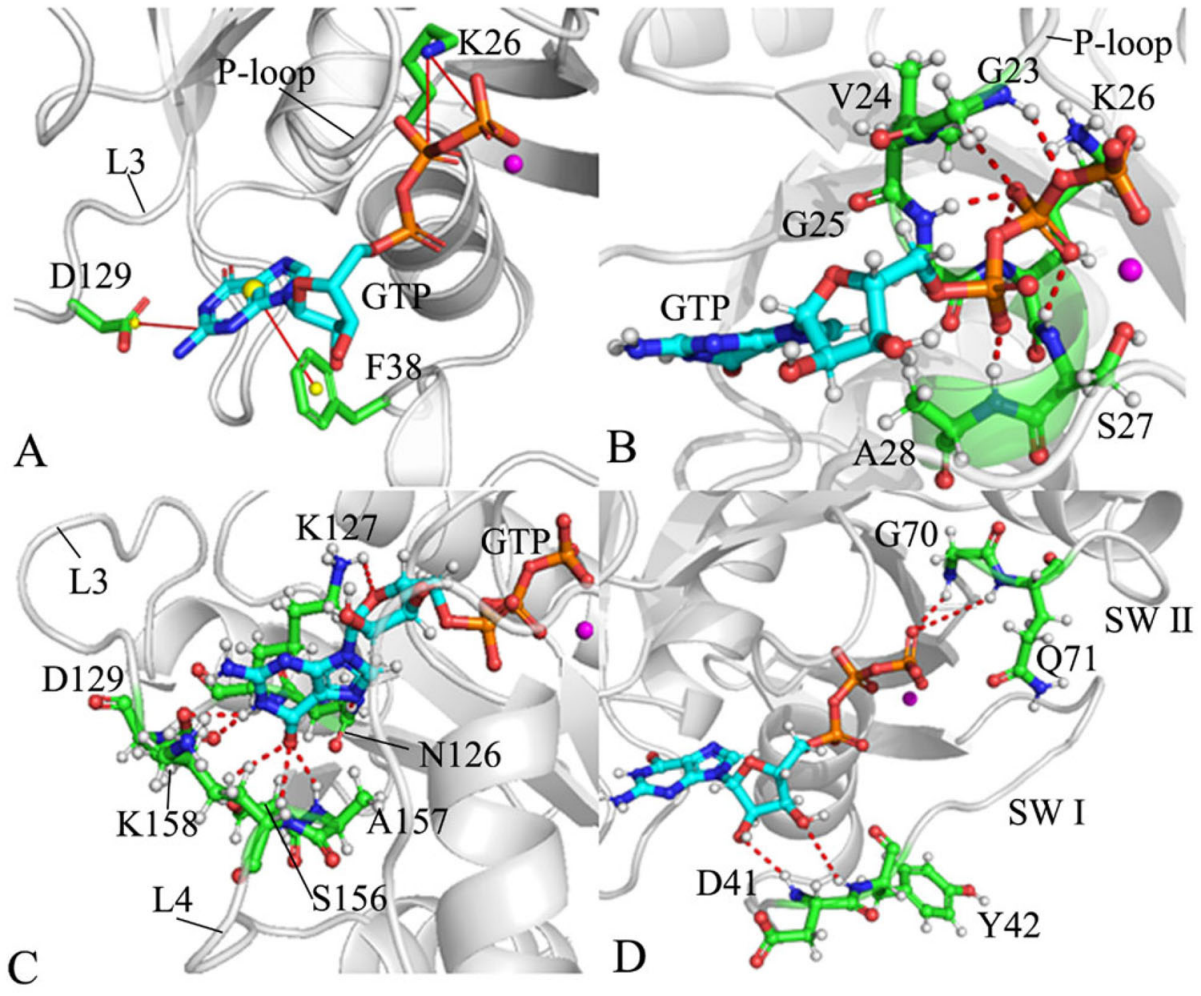
Geometric positions of GTP relative to key residues of M-RAS: (A) the salt bridge interactions of GTP with K26 and D129 as well as the π–π interaction of GTP with F38, (B) HBIs of GTP with residues in the P-loop, (C) HBIs of GTP with residues in the loops L3 and L4 and (D) HBIs of GTP with residues in the switch domains.

The previous analyses of FELs reveal the changes in interactions of GTP with M-RAS caused by conformational transitions. To evaluate the stability of the interaction network between GTP and M-RAS, the distances of non-bonded salt bridge interactions and the π-π interactions of GTP with M-RAS were calculated and the results were displayed in Figure 9 and Figure S10. The HBIs of GTP with M-RAS were also analysed by using the program CPPTRAJ in Amber 20 and the information was listed in Table 1. The geometric positions involved in the aforementioned interactions were displayed in Figure 10 by means of the most compact structure of the GTP-bound WT M-RAS.

**Table 1.** Hydrogen bonding interactions of GTP with the WT and mutated M-RAS.

^a^Hydrogen bonds		^b^Occupancy(%)			
M-RAS residues	GTP atoms	WT	P40D	D41E	P40D/D41E/L51R
G23-N-H	O3B	92.8	92.4	91.5	90.8
V24-N-H	O1B	21.6	20.7	12.5	13.8
G25-N-H	O1B	97.7	97.9	99.5	99.4
K26-N-H	O1B	99.9	99.9	99.9	99.9
S27-N-H	O2B	99.7	99.7	95.9	94.2
A28-N-H	O1A	99.8	99.7	99.8	99.8
D40-OD1	O2'-HO'2	0	11.1	0	0
D41-N-H	O2'	11.2	9.8	0	0
Y42-N-H	O3'	15.6	2.5	2.2	1.1
G70-N-H	O1G	85.3	79.9	5.6	12.1
Q71-N-H	O1G	4.5	3.1	5.3	1.8
N126-ND2-HD21	N7	83.3	82.7	88.3	88.1
K127-NZ-HZ1	O4'	21.2	25.4	39.6	36.9
D129-OD1	N1-H1	89.4	90.6	56.2	48.4
D129-OD2	N1-H1	80.8	79.9	67.5	44.1
S156-OG-HG	O6	54.3	55.8	56.1	55.3
A157-N-H	O6	90.7	89.8	92.2	90.2
K158-N-H	O6	45.5	44.9	41.2	43.3

^a^Hydrogen bonding interaction are recognised by an acceptor·donor distance of <3.5 Å and acceptor·H-donor angle of >120°.

^b^Occupancy (%) is defined as the percentage of simulation time that a specific hydrogen bond exists.

According to the results revealed by the PLIP sever, residue K26 in the P-loop produces two salt bridge interactions with the phosphate group of GTP (Figure 10(A)). To check effect of three mutations on these two salt bridges, the distances of the nitrogen atom NZ of K26 away from the phosphorus atoms PB and PG of GTP were calculated (Figure 9(A) and S^10^(A)). The distance between the NZ and PB fluctuates from 3.0 to 5.3 Å through the entire GaMD simulations of four systems (Figure 10(A)), verifying that the relative position of K26 to the phosphate group is stable. This distance in the GTP-bound D41E and P40D/D41E/L51R M-RAS is reduced by 0.2 Å relative to that in the GTP-bound WT and P40D M-RAS (Figure 9(A)), showing that D41E and P40D/D41E/L51R strengthens the salt bridge interaction between the NZ and PB while P40D hardly impacts the strength of this salt bridge. The distance between the NZ and PG is located at a range from 3.1 to 6.2 Å during GaMD simulations of four systems (Figure S10(B)), on the whole, this distance is stable. The distance between the NZ and PG is situated at the peak value of 3.8 Å in four systems (Figure 9(B)), indicating that these three mutations do not produce influences on the salt bridge interactions between the NZ and PG. As shown in Figure 10(A), the carbonyl group of D129 in the loop L3 forms a salt bridge interaction with the guanine group of GTP. To check the effect of mutations on this salt bridge, the distance between the mass centre of the oxygen atoms OD1 and OD2 of D129 and the mass centre (at the carbon atom C2) of the nitrogen atoms N1-N3 of GTP were computed and their time evolutions were exhibited Figure S10(C). This distance between two mass centres fluctuates from 3.1 to 4.9 Å, which shows high stability. As observed in Figure 9(C), the distance of the salt bridge interaction between D129 and GTP is located at the peak position of 3.5 Å in four systems, indicating that mutations hardly exert influences on the salt bridge interaction of D129 with GTP. In addition, the guanine group of GTP generates a π–π interaction with the phenyl group of F38 (Figure 10(A)). The distance between the mass centres of the guanine group from GTP and the phenyl group of F38 was estimated and its time evolution was provided Figure S10(D). The fluctuation range of the distance corresponding to the π–π interaction is located at 4.1–11.8 Å, on the whole, this distance is stable during simulations. More importantly, the frequency distribution of this π-π interaction almost overlaps in four systems (Figure 9(D)), implying that mutations hardly produce impacts on this π-π interaction between GTP and F38.

According to Figure 10(B), the phosphate group of GTP forms HBIs with the conserved residues G23, V24, G25, K26, S17 and A28 in the P-loop and the occupancy of these hydrogen bonds is greater than 90.8% apart from residue V24 (Table 1), suggesting that these HBIs are highly stable during GaMD simulations. Compared to the WT M-RAS, except for V24, three mutations do not generate a great effect on those HBIs of GTP with the residues from the P-loop, which provides rational explanation for the high stability of the P-loop in M-RAS during GaMD simulations. As displayed in Figure 10(C), the guanine group of GTP produces HBIs with residues N126, K127 and D129 in the loop L3 and residues S156, A157, and K158 in the loop L4. Except for K127, the occupancy of HBIs of GTP with the other residues in the L3 and L4 is higher than 44.1% (Table 1), indicating that these hydrogen bonds play an important role in the binding of GTP to M-RAS. By comparison with the WT M-RAS, P40D hardly affects the stability of HBIs of GTP with residues in the L3 (Table 1). Meanwhile, three mutations also hardly impact HBIs of GTP with the residues in the L4 (Table 1). Although D41E and P40D/D41E/L51R increase the occupancy of hydrogen bond between GTP and N126 compared to the GTP-bound WT M-RAS, they decrease that of HBIs of GTP with K127 and D129 (Table 1). The middle ring in GTP forms HBIs with two residues D41 and Y42 from the SW I and the occupancy of these two hydrogen bonds is smaller than 15.6% (Table 1 and Figure 10(D)), reflecting that these two hydrogen bonds are extremely instable. This result clarifies the reason why the SW I is highly flexible and disordered. Meanwhile, P40D directly brings an HBI of GTP with D40 in the GTP/P40D M-RAS complex, but this hydrogen bond is instable. Figure 10(D) shows that the phosphate group of GTP generates two HBIs with residues G70 and Q71 in the SW II. The occupancy of the hydrogen bond between GTP and Q71 in four systems is lower than 5.3% (Table 1), verifying this hydrogen is extremely instable. By referencing the WT M-RAS, the occupancy of the hydrogen bond between GTP and G70 is decreased by 5.4, 79.7 and 73.2% because of P40D, D41E, and P40D/D41E/L51R (Table 1), respectively, which affects the conformational changes of the loop in the SW II. The work from Shima et al. also revealed that the alterations in interactions of T45, G70 and Q71 with GTP induced by mutations play an important role in the state transition of the switch domains^19^, which supports our findings. The study from Ye et al. unveiled that the instability in interactions of GDP with D41 and Y42 greatly impacts the conformational changes of the switch domains in M-RAS^27^, which is in agreement with our work.

Based on the above analyses, it is found that the salt bridge interactions of GTP with K26 and D129 and the π–π interaction of GTP with F38 play a vital role in the binding of GTP to M-RAS, which is favourable for the stability of the P-loop and the loop L3. Meanwhile, the high instability in HBIs GTP with residues 41 and Y42 in the SW I and residue G70 in the SW II of M-RAS is responsible for the disorder states of the switch domains, which is supported by the previous work^19^^,^^27^. It is well known that the SW I mostly overlaps with binding regions of M-RAS to effectors, thus HBIs of GTP with D41 and Y42 are significant for the activity of M-RAS.

## Conclusions

Conformational changes of M-RAS caused by mutations are involved in the development of human cancers. To clarify molecular mechanism with regard to the conformational transition of M-RAS, 4.8-μs GaMD simulations, consisting of four independent replicas and each for running 1.2 μs, were performed on the GTP-bound WT, P40D, D41E, and P40D/D41E/L51R M-RAS to enhance conformational sampling of M-RAS. The calculated RMSFs suggest that P40D, D41E and P40D/D41E/L51R greatly change the structural flexibility of the switch domains and the analyses of DCCMs also demonstrate that three mutations alter the internal dynamics of the switch domains. The constructed FELs verify that mutations induce less energetic states than the WT M-RAS and the switch domains of M-RAS are highly disordered, in particular for the switch domain SW I. The PCA uncover that mutations change collective motions of the switch domains compared to the WT M-RAS. In fact, the SW I is involved in the binding of M-RAS to effectors, hence the highly disordered states of the SW I and the alterations in concerted motions of the SW I must affect the activity of M-RAS. The analyses of the interaction network between GTP and M-RAS using the PLIP server indicate that the high instability in HBIs of residue 41 and Y42 in the SW I drives the high disorder of the switch domains. We expect that this work can contribute to molecular mechanism relating to conformational changes for deeply understanding the function of M-RAS and drug design of anticancer treatment towards the RAS family.

## Supplementary Material

Supplemental Material
