# Supplementary material for: Probing mutation-induced conformational transformation of the GTP/M-RAS complex through Gaussian accelerated molecular dynamics simulations
Source: J Enzyme Inhib Med Chem. 2023 Apr 14;38(1):2195995. doi: 10.1080/14756366.2023.2195995 (PMC10114971; doi:10.1080/14756366.2023.2195995)
Supplement: Supplemental Material [file IENZ_A_2195995_SM8928.pdf]

Probing mutation-induced conformational transformation of the GTP/M-RAS complex through Gaussian accelerated molecular dynamics simulations

Huayin Bao[1], Wei Wang[b], Haibo Sun[b] and Jianzhong Chen[b]

[a]School of Pharmacy, Shandong University of Traditional Chinese Medicine, Jinan, China 250355;

[b]School of Science, Shandong Jiaotong University, Jinan, China 250357

CONTACT Huayin Bao E-mail: huayinbao@126.com School of Pharmacy, Shandong University of Traditional Chinese Medicine Jinan, China; Jianzhong Chen E-mail: chenjianzhong1970@163.com; jzchen@sdjtu.edu.cn School of Science, Shandong Jiaotong University Jinan, China.

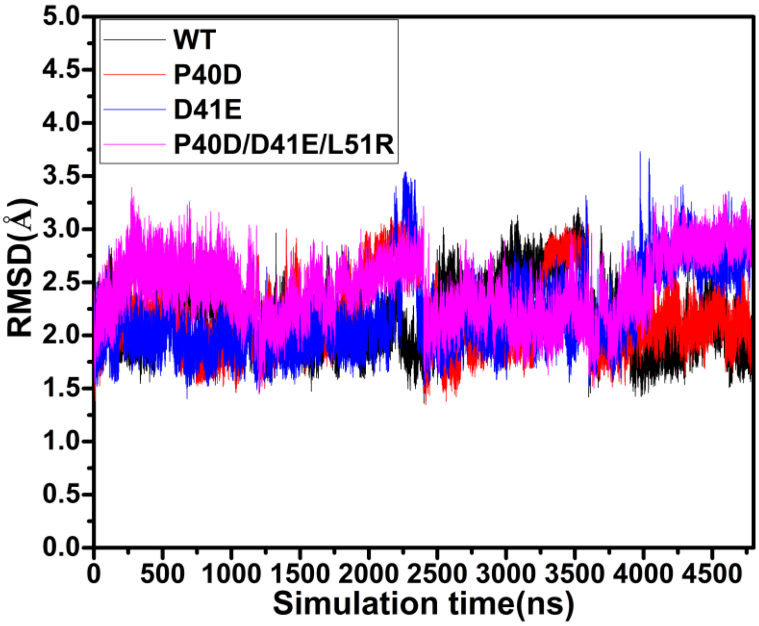

Figure S1. RMSDs of non-hydrogen atoms from the WT and mutated M-RAS calculated relative to the initial minimized structures.

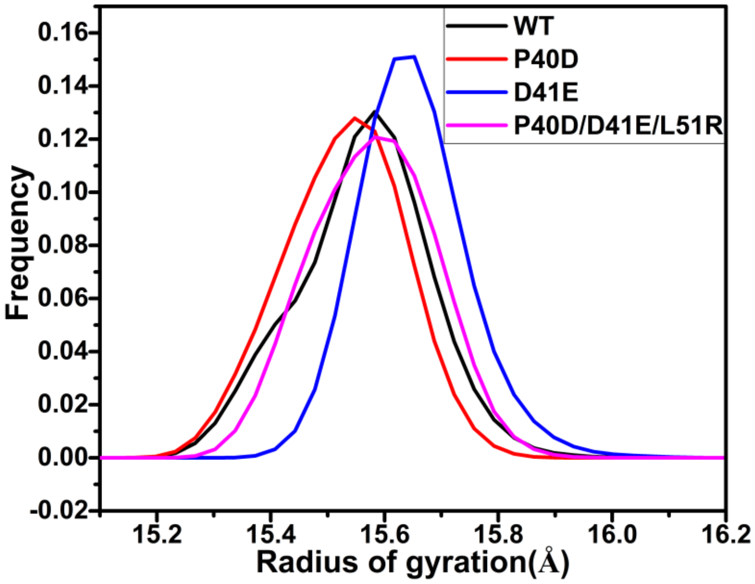

Figure S2. Frequency distribution of radius of gyration for the GTP-bound WT and mutated M-RAS.

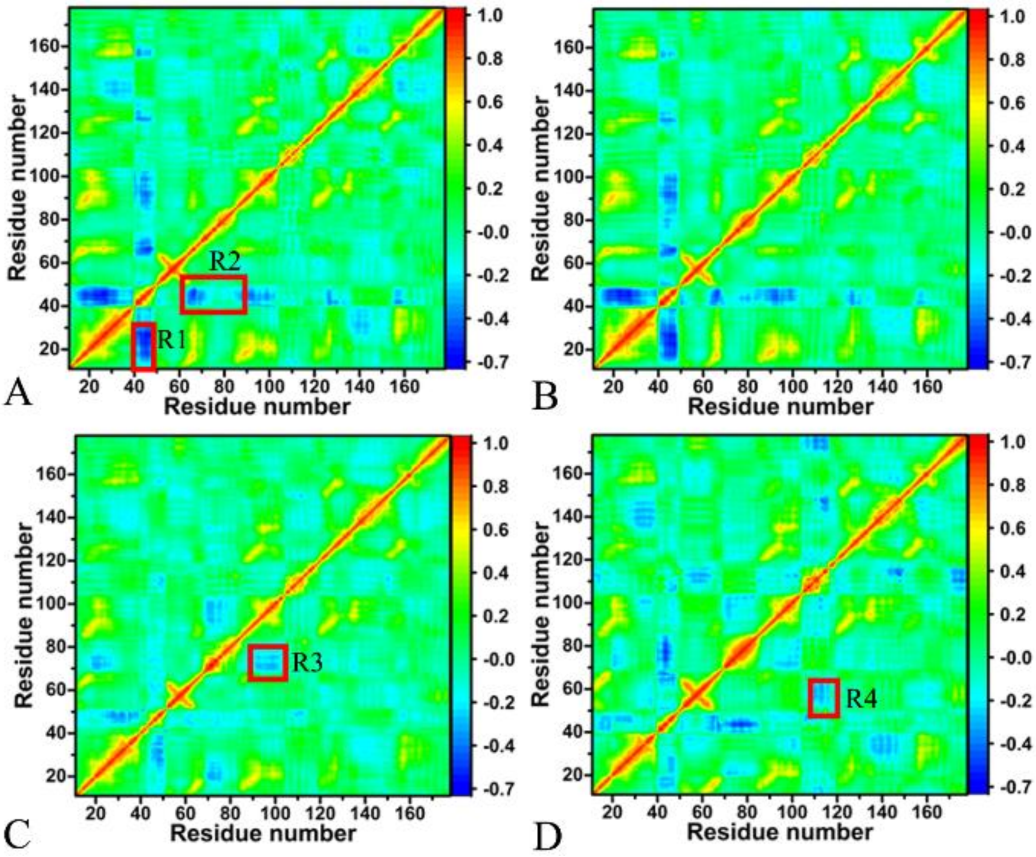

Figure S3. Correlated motions between residues of M-RAS reflected by DCCMs: (A) the GTP-bound WT M-RAS, (B) the GTP-bound P40D M-RAS, (C) the GTP-bound D41E M-RAS and (D) the GTP-bound P40D/D41E/L51R M-RAS.

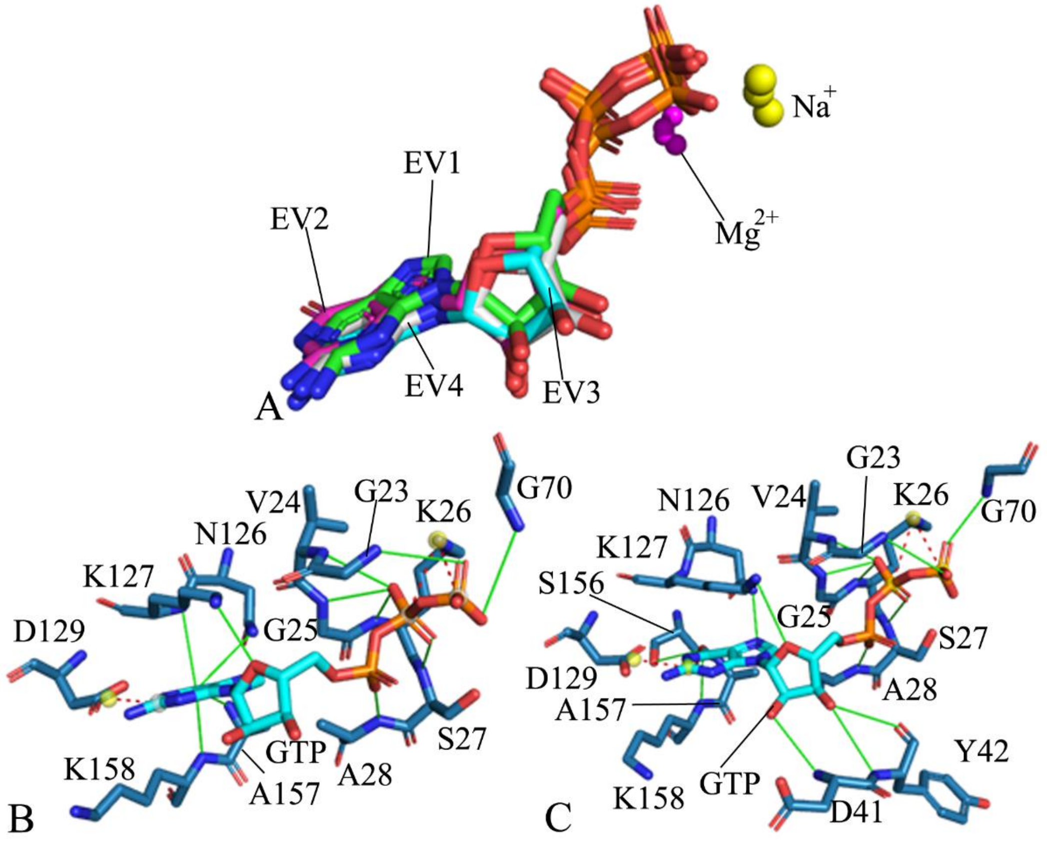

Figure S4. Structural information of GTP with the WT M-RAS: (A) structural superimposition of GTP and magnesium ion located the energy valleys EV1-EV4, (B) GTP-residue interactions in the most incompact state of the switch domains and (C) GTP-residue interactions in the most compact state of the switch domains.

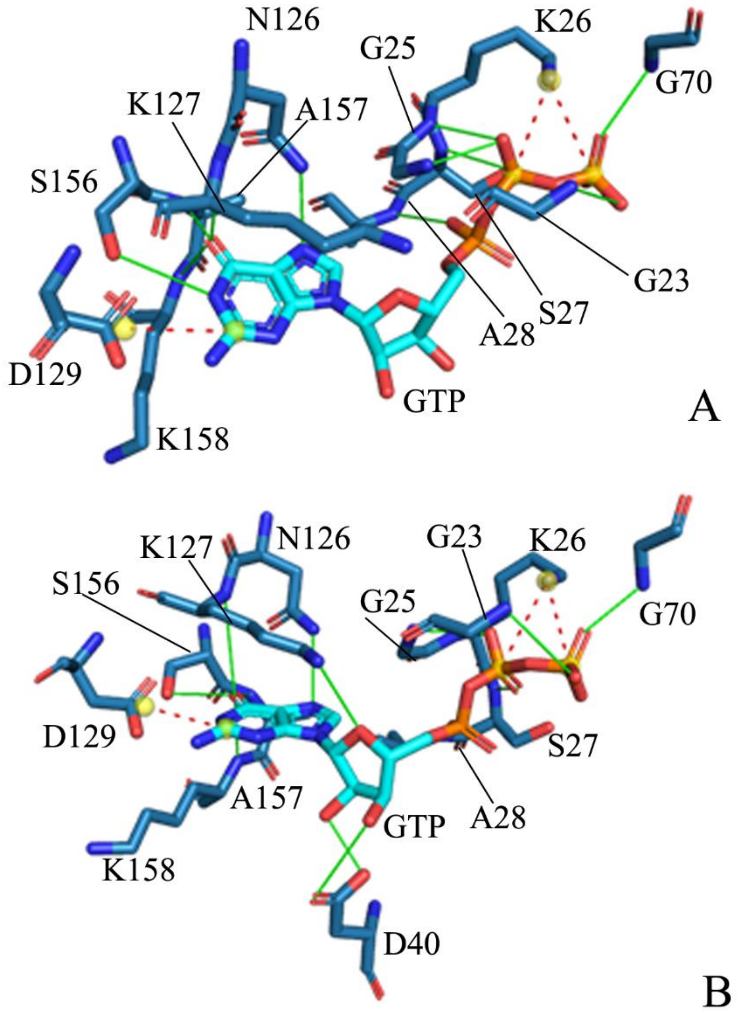

Figure S5. Interaction networks of GTP with the P40D M-RAS in different states of the switch domains: (A) the most incompact state of the switch domains and (B) the most compact state of the switch domains.

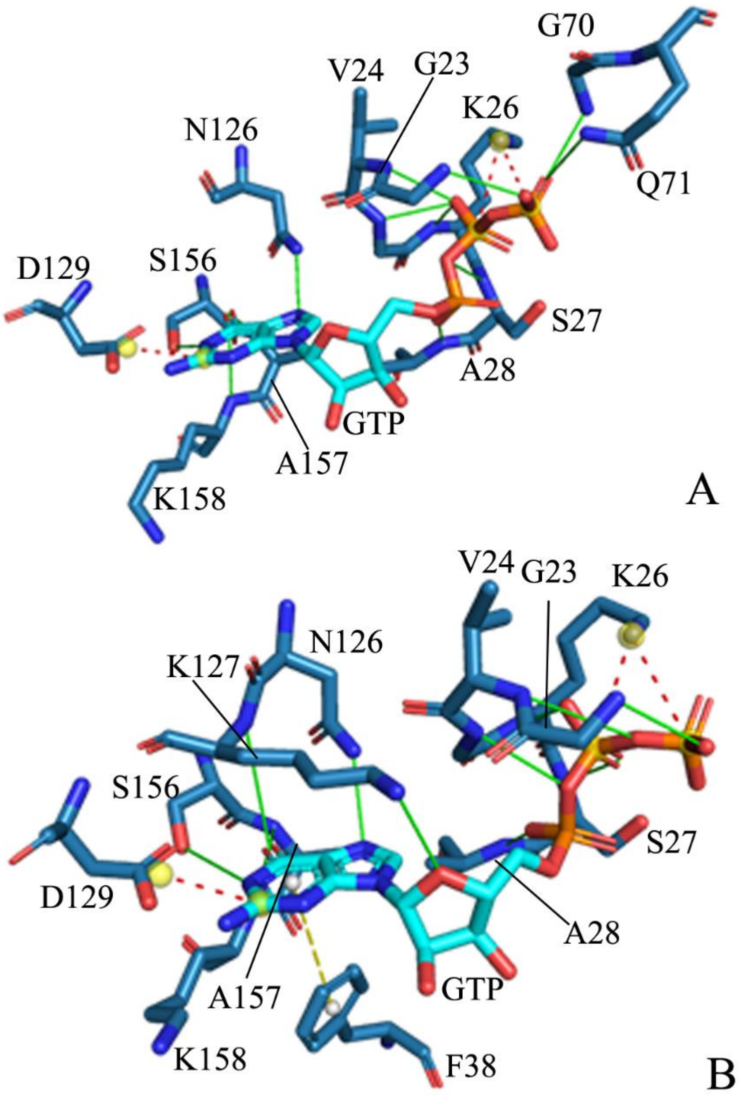

Figure S6. Interaction networks of GTP with the D41E M-RAS in different states of the switch domains: (A) the most incompact state of the switch domains and (B) the most compact state of the switch domains.

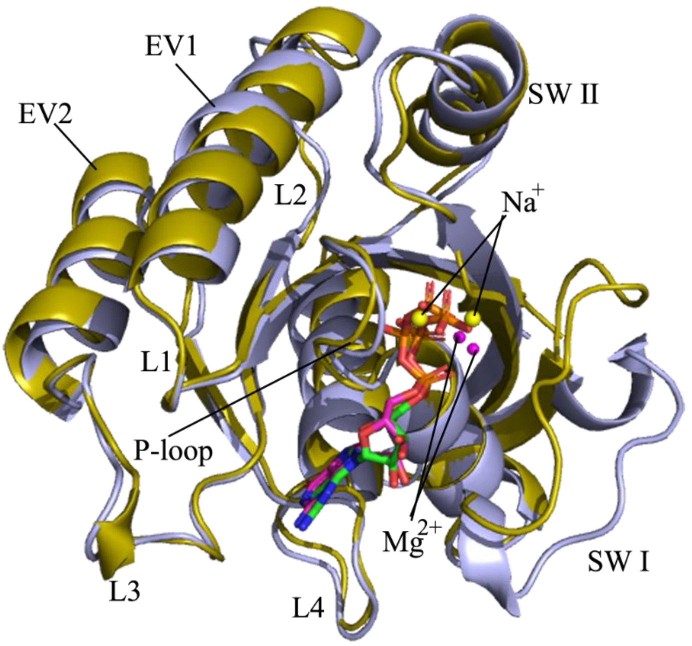

Figure S7. Structural superimposition of the GTP-bound P40D/D41E/L51R M-RAS situated at the EV1 and EV2.

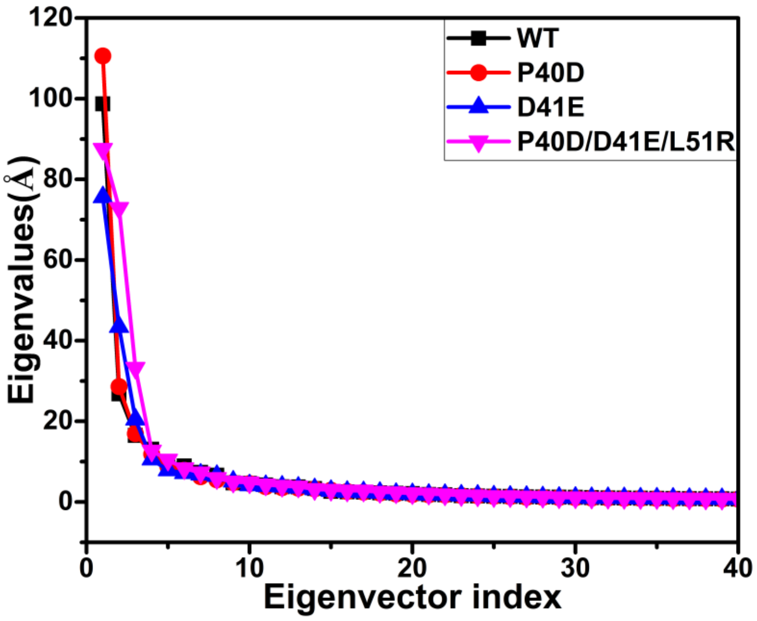

Figure S8. Function of eigenvalues VS eigenvector indexes from principal component analysis.

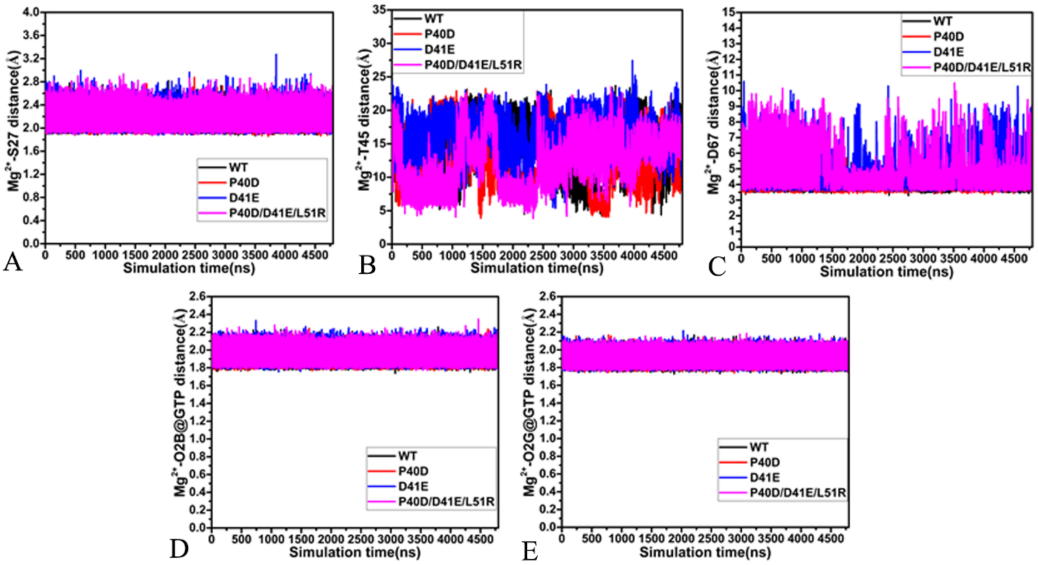

Figure S9. The time evolution of the distance for Mg^{2+} interactions: (A) the time evolution of the distance for Mg^{2+} away from the oxygen atom OG of S27 in the P-loop of M-RAS, (B) the function of the distance between Mg^{2+} and the oxygen atom O of T45 in the SW I of M-RAS as the simulation time, (C) the time evolution of the distance for Mg^{2+} away from the mass center of the oxygen atoms OD1 and OD2 from D67 in the SW II of M-RAS, (D) the function of the distance between Mg^{2+} and the oxygen atom O2B of GTP as the simulation time and (E) the time evolution of the distance for Mg^{2+} away from the oxygen atom O2G of the GTP.

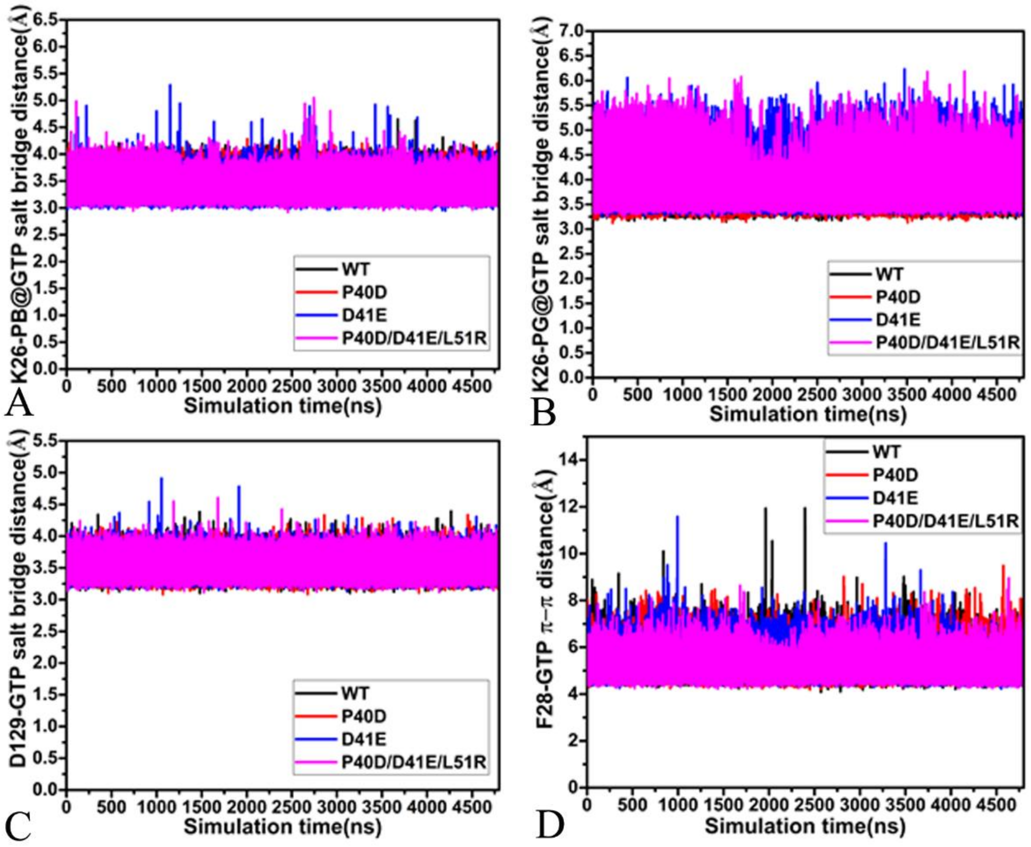

Figure S10. (A) the time evolution of the distance for the nitrogen atom NZ of K26 away from the phosphorus atom PB of GTP, (B) the time evolution of the distance between the nitrogen atom NZ of K26 and the phosphorus atom PG of GTP, (C) the time evolution of the distance for salt bridge interaction of the carbonyl of D129 with the guanine group of GTP and (D) the time evolution of the distance for the π-π interaction of the phenyl group of F28 with the guanine group of GTP.
